# Integrated improved Harris hawks optimization for global and engineering optimization

**DOI:** 10.1038/s41598-024-58029-3

**Published:** 2024-03-28

**Authors:** Chengtian Ouyang, Chang Liao, Donglin Zhu, Yangyang Zheng, Changjun Zhou, Taiyong Li

**Affiliations:** 1 School of Information Engineering, Jiangxi University of Science and Technology, Ganzhou, 341000 China; 2 School of Computer Science and Technology, Zhejiang Normal University, Jinhua, 321004 China; 3 School of Computing and Artificial Intelligence, Southwestern University of Finance and Economics, Chengdu, 611130 China

**Keywords:** Integrated improved Harris hawks optimization, Intermittent energy regulator, Levy flight, Attenuation vector, Cardano formula, Engineering, Mathematics and computing

## Abstract

The original Harris hawks optimization (HHO) algorithm has the problems of unstable optimization effect and easy to fall into stagnation. However, most of the improved HHO algorithms can not effectively improve the ability of the algorithm to jump out of the local optimum. In this regard, an integrated improved HHO (IIHHO) algorithm is proposed. Firstly, the linear transformation escape energy used by the original HHO algorithm is relatively simple and lacks the escape law of the prey in the actual nature. Therefore, intermittent energy regulator is introduced to adjust the energy of Harris hawks, which is conducive to improving the local search ability of the algorithm while restoring the prey's rest mechanism; Secondly, to adjust the uncertainty of random vector, a more regular vector change mechanism is used instead, and the attenuation vector is obtained by modifying the composite function. Finally, the search scope of Levy flight is further clarified, which is conducive to the algorithm jumping out of the local optimum. Finally, in order to modify the calculation limitations caused by the fixed step size, Cardano formula function is introduced to adjust the step size setting and improve the accuracy of the algorithm. First, the performance of IIHHO algorithm is analyzed on the Computational Experimental Competition 2013 (CEC 2013) function test set and compared with seven improved evolutionary algorithms, and the convergence value of the iterative curve obtained is better than most of the improved algorithms, verifying the effectiveness of the proposed IIHHO algorithm. Second, the IIHHO is compared with another three state of the art (SOTA) algorithms with the Computational Experimental Competition 2022 (CEC 2022) function test set, the experiments show that the proposed IIHHO algorithm still has a strong ability to search for the optimal value. Third, IIHHO algorithm is applied in two different engineering experiments. The calculation results of minimum cost prove that IIHHO algorithm has certain advantages in dealing with the problem of search space. All these demonstrate that the proposed IIHHO is promising for numeric optimization and engineering applications.

## Introduction

Meta heuristic technology is a product derived from the field of mathematics, the research progress in the field of meta heuristic is often applicable to the behavior of human life and production. Taking particle swarm optimization (PSO) algorithm as an example, Wang et al.^1^ designed a new potential aware fuzzy scheme, namely syntony (SYNTONY), which uses the efficient PSO to select effective seeds, and according to the experiment, SYNTONY significantly increases the edge coverage; Liu et al.^2^ proposed a migration based PSO algorithm, which uses dynamic differential grouping for online decomposition. Experiments show that the solution of the proposed algorithm is superior to the current most advanced algorithm in the case of problems up to 1000 dimensions; Raghav^3^ proposed an improved PSO (mPSO) algorithm to solve the economic scheduling problem. The experimental results show that the proposed algorithm has a more reasonable economic cost; Zhang et al.^4^ proposed a multi-objective collaborative PSO (MOCPSO), which combines diversity strategy (DSS) and convergent search strategy (CSS) to increase the local search range of particles, and uses a three category framework to effectively use DSS and CSS; Zhang et al.^5^ proposed an improved discrete PSO (DPSO) algorithm to guide the underwater nozzle array layout of the artificial upflow system. The experiment found that with the increase of iteration times, the layout optimized by DPSO algorithm was more reasonable; Parastoo et al.^6^ proposed a multi-objective discrete PSO (CBMODPSO) based on crossover, which was applied to the parameter evaluation of Multimodal routing problem. The experiment found that the error rate and spacing measurement obtained by using CBMODPSO algorithm were smaller, which proved that the convergence of the algorithm was better; Zhu et al.^7^ proposed a Multi strategy PSO based on Exponential noise (MEPSO) to solve the problem of low altitude penetration in safe space. The experiment found that the proposed algorithm can plan a simpler and clearer path in different complex environments; Zhu et al.^8^ also proposed an improved bare skeleton PSO (IBPSO), which was applied to solve the problem of DeoxyriboNucleic Acid (DNA) design. The experiment found that the DNA sequence designed by the proposed algorithm can avoid the secondary structure and effectively reduce the value of h-measure and similarity combination constraint. Besides PSO algorithm, other algorithms also have certain advantages.

Other algorithms have also achieved better experimental results in specific problems through improvement or combination, which proves the research value of the improvement. Zhu et al.^9^ proposed a human memory algorithm (HMO), and the experiment found that the proposed algorithm can get smaller optimal value. Zhao et al.^10^ proposed the improved gray wolf algorithm (IGWO), and the simulation experiment found that the improved gray wolf algorithm has good consistency. Zamani and Nadimi-Shahraki^11^ proposed an evolutionary crow search algorithm (ECSA) to optimize the hyperparameters of ANNs for diagnosing chronic diseases,and the experiment indicated the superiority of ECSA over competitor algorithms in optimizing the network. NadimiShahraki et al.^12^ proposed a new binary optimizer algorithm (BSMO), which is based on the newly proposed starling murmuration optimizer (SMO) and use four targeted medical datasets to evaluate the performance of BSMO, the experiment shows satisfactory results in selecting effective features from the targeted medical datasets. Zhu et al.^13^ proposed a Jaya (JAYA) algorithm based on normal cloud, which was applied to the optimization of DNA sequence design and found that the proposed algorithm can effectively control the secondary structure or hybridization in the process of DNA reaction. Xue et al.^14^ proposed a population-based optimization algorithm for dung beetles, which was applied to three well-known engineering design problems. Experiments showed that the proposed algorithm can effectively deal with constraint problems; Zhu et al.^15^ proposed an improved manta ray foraging optimization algorithm MGL-MRFO. Experiments found that using MGL-MRFO algorithm in different environments could ensure good learning ability and adaptability to find reasonable and feasible solutions. Hisham^16^ suggest combining gravitational search algorithm (GSA) and sperm swarm optimization (SSO) to propose a hybird algorithm called HSSOGSA, the experiment found that HSSOGSA will be able to search and explore any search space domain with a fast convergence rate without tripping in a local minimum. Xu et al.^17^ proposed an integrated clustering scheme to Fuse both the global Structure and the local structure information for Ensemble Clustering (FSEC), and used the alternating direction multiplier method (ADMM) to solve the objective function optimization problem. The experiment found that the proposed FSEC was superior to many of the most advanced integrated clustering methods.

Harris hawks optimization algorithm (HHO) is a new biological optimization algorithm proposed by Heidari et al.^18^. It is also one of the algorithms frequently selected in recent years to solve optimization problems. Through in-depth research, scholars have improved and clustered the HHO algorithm in varying degrees, so as to solve the shortcomings of the algorithm itself, such as poor search performance and easy to fall into local optimization in the later phase of optimization, and applied it to a variety of complex engineering problems. Xie et al.^19^ proposed a data-driven method based on HHO genetic programming (HHO-GP), which was applied to 12 prediction models of underground structure life under sulfate corrosion. The results showed that the average relative training error and prediction error of the new prediction model were small. Asad et al.^20^ proposed to combine HHO algorithm with vehicle ad HOC network (VANET) to form a new Clustering algorithm (HHOCNET). Comparing the proposed algorithm with other clustering algorithms, it was found that HHOCNET has the least number of vehicle cluster heads in the whole network, representing the large coverage of each vehicle, proving that the proposed algorithm has higher reliability. Sana et al.^21^ proposed to further optimize the extraction of kernel sharply value (kSV) using HHO algorithm. This kSV-HHO cancer classification method provides the potential to improve interpretability, enhance performance and improve the efficiency of cancer classification. Zhang and Bao^22^ proposed a HHO phase division method based on hard sequence constraints to automatically determine the optimal number of phases. The advantage of this HHO based phase division is that it can successfully find the optimal number of phases according to the percentage of performance improvement indicators. In recent years, the application of leader HHO has become more and more frequent. Ayinla et al.^23^ proposed using IHHO to design proportional integral derivative (PID) and fractional order proportional integral derivative (FOPID) controllers to realize the optimal speed regulation of direct current (DC) motor. Experiments show that the proposed controller has significantly improved the rise time, stability time and the maximum overshoot during transient. Bibhuti^24^ proposed to use improved HHO (IHHO) and Taguchi coupling additive ratio assessment (ARAS) technology to study the optimal cutting conditions and variables affecting the cutting parameters of titanium alloy. At present, it has been used to minimize tool wear, chip reduction coefficient and surface roughness. Liu et al.^25^ proposed a multi leader HHO model with adaptive mutation(MIHHO-AM), and used the algorithm to optimize the parameters of Elman neural network (ENN). The experiment found that the proposed model has higher accuracy in predicting the silicon content in blast furnace molten iron and can better adapt to the changing trend.

The improved HHO algorithm is also applied in information technology, electrical power, medical and other fields. Khatri et al.^26^ proposed the discrete natural inspired HHO algorithm DHHO, which was applied to the independent cascade model of information diffusion to evaluate the effectiveness of eight social networks. The experiment found that DHHO has a higher final impact in almost all data sets. Gharehchopogh et al.^27^ proposed a new binary multi-objective dynamic HHO algorithm (MODHHO) based on mutation operator, which was applied to identify botnets in the Internet of things. The experiment found that the use of MODHHO algorithm can show low error rate and high accuracy in the optimization operation. Hussein et al.^28^ proposed a boosted HHO algorithm (BHHO), which was applied to the problem of extracting the parameters of single diode peak voltage (PV). The experimental data under seven weather conditions were used to verify the performance of the algorithm. The experiment proved that the proposed algorithm has high consistency and converges to the optimal under all environmental conditions. Ebrahim et al.^29^ proposed an optimization technology combining HHO and sine cosine algorithm (SCA), which was applied to determine the parameters of the optimal control DC bus voltage controller. The experiment found that the proposed algorithm improved the DC bus voltage and battery response, and improved the overall efficiency and fuel cell life. Zhou and Bian^30^ first proposed an multi improved double objective HHO (MBOHHO) algorithm to solve the problem of sustainable robot disassembly line balance. The experiment found that the number of Pareto solutions obtained by MBOHHO algorithm was more, and the value of inter-generational distance was lower, which proved that the algorithm had better convergence and better search ability. Aneesh et al.^31^ proposed a new differential evolution adaptive HHO (DEAHHO), which was applied to multi-level image threshold segmentation. The experiment found that although the budget time of using DEAHHO algorithm was more, the fitness value was better. Zhang et al.^32^ proposed a SSFSHHO that integrates Sobol sequence and random fractal search (SFS) mechanism to classify Alzheimer's disease (AD) and early mild cognitive impairment (MCI). Experiments show that this algorithm is superior to many classical machine learning algorithms and improves the classification performance of AD diagnosis to a certain extent.

The above improved HHO algorithms are obviously superior to the original algorithm in their respective original texts, but first of all, the improved HHO algorithms also have problems such as unstable optimization effect, easy to fall into stagnation, and high standard deviation when dealing with high-dimensional problems. Second, the actual changes of Harris hawk and prey in nature are often ignored in the design of the improvement strategy. Finally, the basic mathematical structure principles are not thoroughly quoted in the process of adding innovation points, Therefore, it is necessary to modify the algorithm and obtain a new improved version. While solving the shortcomings of the original HHO algorithm in the research, we can get an algorithm with higher versatility, stability and further improved optimization effect. The main contributions of this study are as follows:

1. A new integrated improved Harris hawks optimization algorithm (IIHHO) is proposed. Firstly, the intermittent energy adjustment factor is used to affect the nonlinear change of energy. The real intermittent energy regulator can effectively improve the local search ability of the algorithm. Then, combined with the compound function formula, the random vector of Levy flight is modified to obtain the attenuation vector to obtain a more accurate search range, which is conducive to jumping out of the local optimum. Finally, by setting Cardano formula function to adjust the step size, the accuracy of the algorithm is improved.
2. The solution generated by IIHHO in the 50 dimensional test function is evaluated by using the computational experiment competition 2013 (CEC 2013) test suite. Firstly, the ablation experiment is used to verify the effectiveness of each innovative mechanism. Secondly, compared with other improved algorithms, it is determined that the optimal value and other data indicators obtained by the improved algorithm in most functions are optimal. Finally, in the computational experiment competition 2022 (CEC 2022), the proposed IIHHO algorithm is compared with three latest SOTA algorithms to verify the effectiveness of the algorithm.
3. The algorithm is applied to two engineering experiments of welded beam design and pressure vessel design. The experiment shows that the parameter optimization performance of IIHHO algorithm is better, and the cost consumption value of 5884.74947 is smaller than that of other algorithms in solving pressure vessel problems, which verifies the performance of the IIHHO algorithm.

The remainder of this paper is organized as follows: The basic steps of the original HHO algorithm are presented in the sections of “Harris hawks optimization algorithm” section. “Integrated improved Harris hawks optimization algorithm” section focuses on the improvement strategy of HHO algorithm. In “Experiments” and “Performance of IIHHO on engineering applications” sections, the experiment of the proposed method is carried out, and the results are demonstrated and analyzed. Based on the experimental results, the obtained algorithm IIHHO and other algorithms are analyzed and summarized at the same time. “Conclusion” section is a elaboration and induction of the existing work and future actions and expectations.

## Harris hawks optimization algorithm

This section mainly describes the initialization phase, exploration phase, transition phase and development phase of HHO algorithm.

### Initialization phase

In the initialization phase of the algorithm, the position and fitness value of each individual in the population are initialized by random generation. The fitness value is constantly updated through the objective function in the iteration process, so the objective function for solving the fitness value also needs to be initialized. At the same time, all parameters in the function need to be set with corresponding boundary constraints.

### Exploration phase

In the HHO model, the exploration phase is a link that simulates the Harris hawk's random stay strategy mechanism, sets the random selection factor ${q}_{r}$ of the value range [0,1], and in each iteration, takes the probability value of 0.5 as the boundary, and carries out different location selection strategies according to the size of ${q}_{r}$. When ${q}_{r}$≥0.5, the Harris hawk needs to select a random location to explore, while when ${q}_{r}$<0.5, The location strategy of Harris hawk is to choose the location of other family members. Its mathematical expression is as follows:

1 $$X(t+1)=\left\{\begin{array}{ll}{X}_{rand}(t)-{r}_{d1}|{X}_{rand}(t)-2{r}_{d2}X(t)| & \quad {q}_{r}\ge 0.5\\ {(X}_{p}(t)-{X}_{m}(t))-{r}_{d3}(lb+{r}_{d4}(ub-lb)) & \quad {q}_{r} < 0.5 \end{array}\right.$$

where ${r}_{d1}$, ${r}_{d2}$, ${r}_{d3}$, ${r}_{d4}$ are random numbers between [0,1]. The updated value in each iteration, *t* is the *t-th* iteration, which affects the position update of the hawk. The parameters $X(t)$, $X(t+1)$, ${X}_{rand}(t)$, ${X}_{p}(t)$ and ${X}_{m}(t)$ in the formula are updated with the value of iteration times *t*, representing the current position vector of the selected hawk, the position vector of the next iteration of the hawk, the position vector of the current randomly selected hawk, the position vector of prey and the average vector of Harris hawk population, *ub* and *lb* respectively represent the upper and lower bounds of variables. For the solution of the average position vector ${X}_{m}(t)$, select the positions of all hawks in the *t-th* iteration for summation, and then take the average value. The corresponding formula is shown in Eq. (2), where the total number of hawks is ${N}_{sum}$:

2 $${X}_{m}(t)=\frac{1}{{N}_{sum}} \sum_{i=1}^{{N}_{sum}}{X}_{i}(t)$$

### Transition phase

The run of sight in the transition stage is determined by the change of prey escape energy. Set the initial stage energy of prey energy as ${E}_{init}$ and update the value during the iteration process within [-1,1]. Set the escape energy ${E}_{time}$ as shown in Eq. (3), where the absolute value is used to judge the implementation of the algorithm in the exploration and development phases, and the critical value 1 shall prevail. When |${E}_{time}$|≥ 1, Harris hawk will carry out the exploration phase, When |${E}_{time}$|< 1, Harris hawk enters the development phase:

3 $${E}_{time}=2{E}_{init} \left (1-\frac{t}{T} \right)$$

### Exploitation phase

After the Harris hawk detects the prey, it can proceed to the development phase. At this time, a new random number *g* is introduced to represent the prey predation probability before the Harris hawk attacks. The value range is [0,1] and the critical value is also 0.5. Finally, the attack strategy used in the development phase is determined through the combination of |${E}_{time}$| and *g*.

#### Soft besiege in the exploitation phase

When *g* ≥ 0.5 and |${E}_{time}$|≥ 0.5, it means that the prey tries to escape but is surrounded by a sudden attack. The corresponding formula is as follows:

4 $$X\left(t+1\right)=\Delta X(t)-{E}_{time}|{B}_{r}{X}_{p}(t)-X(t)|$$

5 $$\Delta X(t)={X}_{p}(t)-X(t)$$

6 $${B}_{r}=2(1-{r}_{d5})$$

where *∆*X(t) is the distance difference between prey and hawk in the *t-th* iteration, ${r}_{d5}$ is a random number between [0,1] and ${B}_{r}$ represents the random jump strength of prey in the process of escape. The value range is [0,2].

#### Hard besiege in the exploitation phase

When *g* ≥ 0.5 and |${E}_{time}$|< 0.5, this state represents the process that the prey itself is more strongly surrounded, exhausted enough after a large amount of energy consumption, and finally captured. The simulation formula is realized as follows:

7 $$X(t+1)={X}_{p}(t)-{E}_{time}|\Delta X(t)|$$

#### Soft besiege with progressive rapid dives

When *g* < 0.5 and |${E}_{time}$|≥ 0.5, Harris hawk will dive. After combining with Levy flight (*LF*) function, the simulation formula is realized as follows:

8 $$Y={X}_{p}(t)-{E}_{time}|B{X}_{p}(t)-X(t)|$$

9 $$Z=Y+S\times LF(dim)$$

10 $$LF\left(x\right)=0.01\times \frac{u\times \sigma }{{\left|v\right|}^{\frac{1}{\beta }}}$$

11 $$\sigma ={\left(\frac{{\Gamma}(1+\beta )\times {\text{sin}}(\frac{\pi \beta }{2})}{{\Gamma}(\frac{1+\beta }{2})\times \beta \times {2}^{(\frac{\beta -1}{2})}}\right)}^{\frac{1}{\beta }}$$

where *dim* is the dimension of the problem, *S* is a random vector with the size of *1* $\times$ *dim*, *LF* is the Levy flight function, and *u* and *v* are random numbers between [0,1], *β* is fixed at 1.5 as the flight step. The updated soft besiege strategy function after introducing *LF* is:

12 $$X(t+1)=\left\{\begin{array}{ll}Y & \quad if \; F(X) < F(X(t))\\ Z & \quad if \; F(Z) < F(X(t))\end{array}\right.$$

#### Hard besiege with progressive rapid dives

When *g* < 0.5, |${E}_{time}$|< 0.5, under the premise of hard besiege, the hawk will shorten its average position interval to capture prey that cannot escape. The simulation formula is:

13 $$X(t+1)=\left\{\begin{array}{ll }Y & if \; F(X)<F(X(t))\\ Z & if \; F(Z)<F(X(t))\end{array}\right.$$

Under the new rules, *Y* and *Z* need to be optimized and adjusted. The simulation formula includes:

14 $$Y={X}_{p}\left(t\right)-{E}_{time}|B{X}_{p}(t)-{X}_{m}(t)|$$

15 $$Z=Y+S\times LF(dim)$$

### Pseudocode of HHO

See algorithm 1 for the pseudocode of HHO algorithm.

**Algorithm 1:**
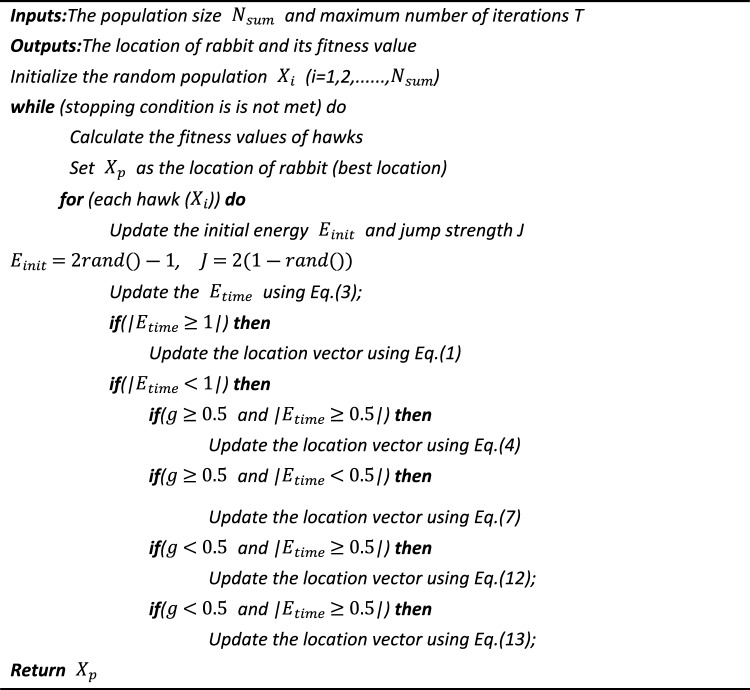
Pseudocode of HHO algorithm.

## Integrated improved Harris hawks optimization algorithm

The basic HHO algorithm still has some shortcomings. First of all, the algorithm has poor search ability in solving problems and is easy to fall into local optimal solution. Secondly, the activity of Harris hawk in natural terrain needs to be considered when constructing HHO model, so HHO algorithm needs to be continuously improved to improve its performance.

### Intermittent energy regulator

The linear decreasing strategy of the original energy of prey in HHO algorithm can not effectively describe the consumption process of the actual prey, and the switching from exploration to development phase of the algorithm is too monotonous, poor balance, and lack of periodic execution operation in the whole iteration process. To address these issues, this paper proposes an intermittent energy adjustment factor for the exploration and development process of phased execution algorithm, And the local search ability of the algorithm is further greatly improved. Accordingly, the original energy formula is modified as follows:

16 $${E}_{nl}=2{{\text{e}}}^{-\left(\uppi \times \frac{{\text{t}}}{{\text{T}}}\right)}$$

17 $$InF={\text{cos}}\left(\frac{2k\mathrm{\pi t}}{{\text{T}}}\right) k=\mathrm{0,1},2,......$$

18 $${E}_{time}=\left\{\begin{array}{ll}{E}_{0}\times {E}_{nl}\times InF & if \; InF\ge 0\\ 0 & if \; InF<0\end{array}\right.$$

**Figure 1:**
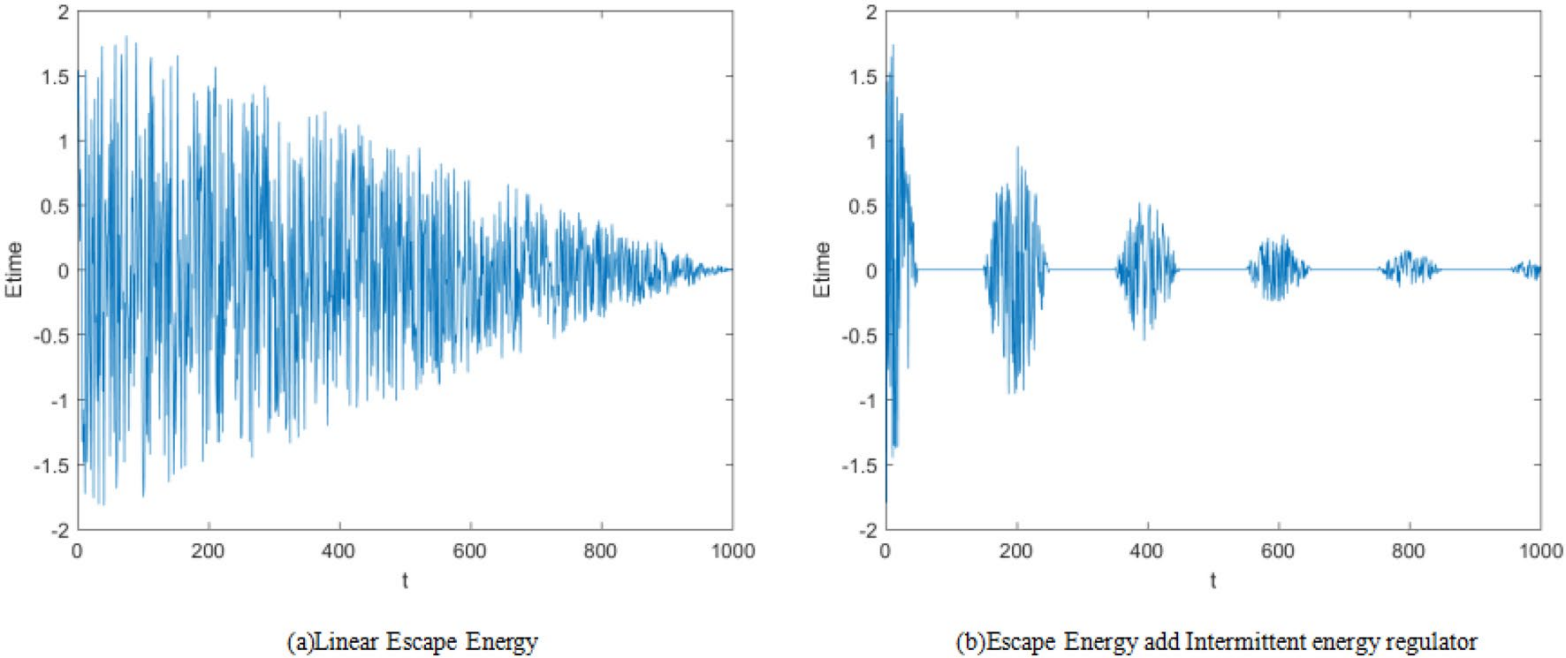
Dynamic comparison diagram of energy change.

The exponential function is used to design the nonlinear energy change ${E}_{nl}$, $\uppi$ is the adjustment coefficient of the value range; Because the cosine function is periodic, the energy is further designed to add cosine disturbance, so as to realize the periodic transformation of energy. The intermittent parameter namely *InF* is designed as shown in Eq. (17).The principle is to multiply the nonlinear energy, further disturb the escape energy, and make intermittent judgement, when *InF* ≥ 0, the energy decreases nonlinearly, while when *InF* < 0, the energy of the prey remains basically unchanged and the displayed value continues to be 0 as shown in Eq. (18), after a period of time, the prey regains activity again to obtain a certain amount of energy, and the algorithm is executed according to this intermittent mechanism. In function (17), *k* = 0,1,2,… indicates the number of decreasing cycles of intermittent parameters. According to the experiment, the effect is the best when *k* = 5. The energy before and after the improvement is as shown in the Fig. 1. After adding intermittent parameters, the algorithm can quickly enter the development phase in the early phase of iteration. With the continuous progress of the cycle, the energy regained by the prey continues to decrease, which the exploration stage is no longer executed, and the time of prey development activities increases significantly, which is conducive to greatly improving the local search ability of the algorithm.

### Attenuation vector

**Figure 2:**
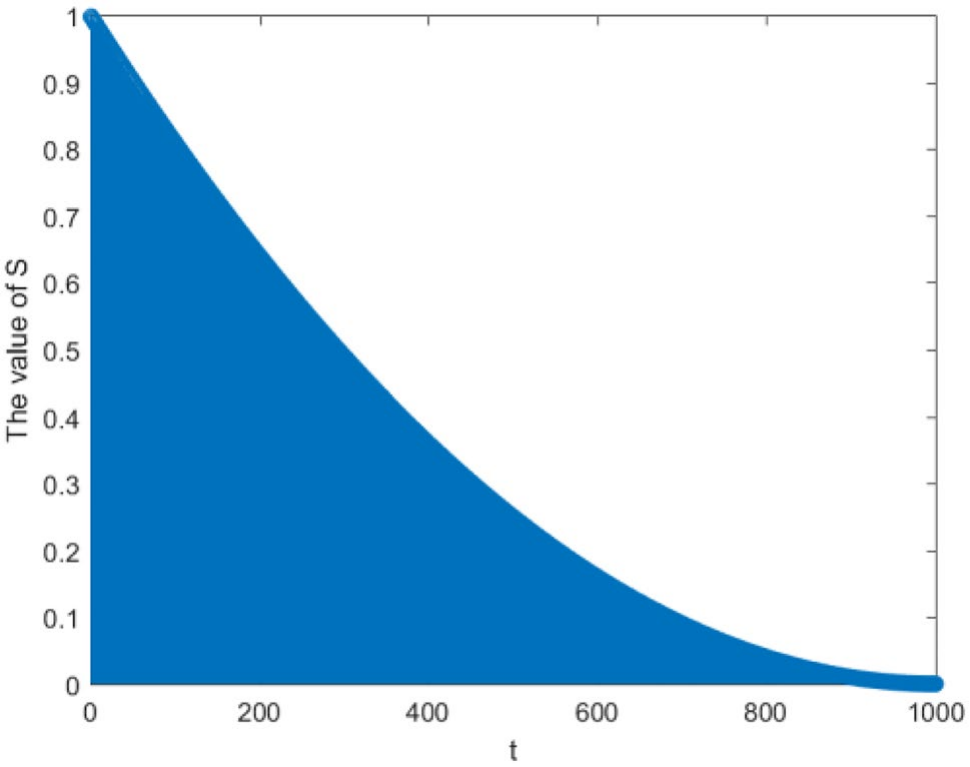
Variation diagram of attenuation vector value.

The original Harris hawk algorithm introduced Levy flight function into the progressive siege form in the development phase. *S*, as an arbitrary *1* $\times$ *dim* vector, randomly affected the position update of Harris hawk under the progressive besiege. Due to the low energy of prey when entering the development phase, the impact of Levy flight should also be reduced in the middle and late phases of iteration, so as to adapt to the implementation activities in the middle and late phases of iteration; By introducing the concept of attenuation, the random vector s is improved, as designed in Eq. (19). The formula uses the principle of monadic quadratic equation, and selects the change curve that the value of the vector gradually decreases with the increase of the number of iterations. Finally, the attenuation effect is realized, which is conducive to further improving the local search ability of the algorithm. The attenuation variation plotted in combination with the design of the function is shown in Fig. 2:

19 $$S={\left(1-\frac{{\text{t}}}{{\text{T}}}\right)}^{2}$$

Combined with the change of attenuation vector, it is found that compared with the original random vector, the improved vector can effectively shorten the average distance between Harris hawk and prey, and use this strategy to generate a new individual position vector, which can help the algorithm jump out of the local optimum and overcome the shortage of premature.

### Step size updating combined with Cardano formula function

The traditional HHO algorithm uses a fixed step value of 1.5. If the step size is too large in the process of Harris hawk searching for prey, there will be no optimal solution. Therefore, in order to make the improved algorithm effectively adjust the search distance, effectively improve the local development ability of HHO algorithm and improve the accuracy of the algorithm, the step size is improved to gradually reduce the large step size at the initial phase of the search to a small step size at the later phase, so as to achieve a smooth transition. Cardano formula is a well-known formula for finding roots of monadic cubic equation. For the monadic cubic equation with standard real coefficients as shown in Eq. (20), one of the function solutions is shown in Eq. (21), where *p* and *q* are known coefficients. When *p* = *q*, the obtained function solution can be regarded as a new monadic cubic function. According to this, this paper updates the flight step size to obtain the Eq. (22) by using the effective function solution obtained by Cardano formula. In order to solve the problem that the population individuals are trapped in a small range at the end of the iteration and stop further iteration, set the critical value of step size, and take 1.2 according to the corresponding value of the experiment. Best, when the step size is less than 1.2, add random value disturbance to the original as shown in Eq. (23), where is the variance after disturbance:

20 $${x}^{3}+px+q=0$$

21 $${x}_{1}=\sqrt[3]{-\frac{q}{2}+\sqrt{\frac{{q}^{2}}{4}+\frac{{p}^{3}}{27}}}+\sqrt[3]{-\frac{q}{2}-\sqrt{\frac{{q}^{2}}{4}+\frac{{p}^{3}}{27}}}$$

22 $$\beta =1.5\times \left(1-\sqrt[3]{-\frac{t}{2T}+\sqrt{\frac{1}{4}\times {\left(\frac{t}{T}\right)}^{2}+\frac{1}{27}\times {\left(\frac{t}{T}\right)}^{3}}}\right)$$

23 $${\sigma }_{c}=\left\{\begin{array}{ll}\sigma & if\; \beta >1.2\\ \sigma \times rand() & if \; \beta \le 1.2\end{array}\right.$$

### Comprehensive review of IIHHO

The IIHHO algorithm is mainly divided into two parts. One is to simulate the intermittent escape energy of prey, and update Eq. (18) through Algorithm 2. The other is to improve the progressive diving mode in the development phase. Compared with other improved HHO algorithms, IIHHO algorithm adjusts the position update formula of progressive subduction and various parameters of levy flight, and changes the random vector into attenuation vector, The original fixed step size is modified by combining with the Cardano function, and the square difference is added with the influence of random number, which completely refined the process operation of the algorithm to jump out of the local optimal solution to a certain extent, and greatly improved the search ability of the algorithm. Because IIHHO algorithm modifies the random vector and fixed step size, the value of the vector and step size in each run of the algorithm is more regular and diversified, which is also conducive to the algorithm to get richer results when solving the individual optimal value, and improve the diversity of the algorithm. See Algorithm 2 for the pseudocode of the proposed IIHHO algorithm.

**Algorithm 2:**
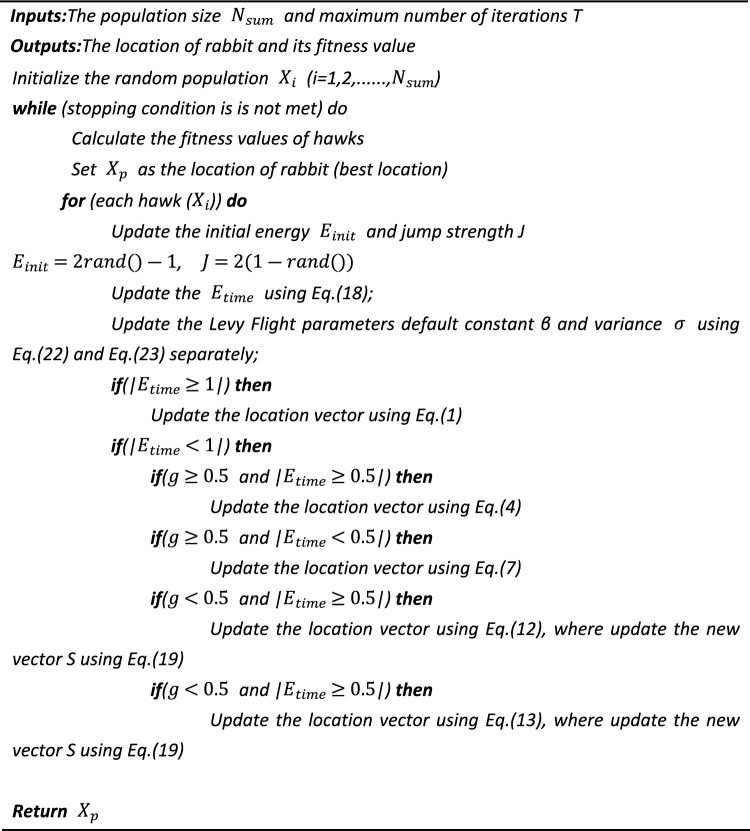
Pseudocode of IIHHO algorithm.

In order to analyze the algorithm more accurately, the time complexity of IIHHO algorithm is further analyzed. In the classical HHO algorithm, the initialization of the swarm of hawks requires O(T × ${N}_{sum}$×*dim*) time, where T represents the maximum number of iterations and has been defined in Chapter 2, meanwhile , *dim* is the dimension of the specific problem. The classical computational complexity of the updated mechanism is O(T × ${N}_{sum}$), so the computational complexity of HHO algorithm is O(T × ${N}_{sum}$×dim) time. Since IIHHO does not supplement any additional process, and the energy update mechanism of IIHHO is modified to intermittent energy regulator, requiring O(T × ${N}_{sum}$) time, the time complexity required after modifying the attenuation vector is O(T × ${N}_{sum}$×*dim*), which is the same as the step size updating combined with Cardano formula function, so there is no difference in cost calculation. In summary, the total computational time for the IIHHO is O(T × ${N}_{sum}$×*dim*) for T iterations.

## Experiments

In this section, an experimental environment is designed to ensure the fairness of the experiment. The computer used for simulation is configured with Intel (R) core (TM) i7-9750h CPU @ 2.60GHz, 8GB ram, 64 bit operating system, and all algorithms are operated in matlab2016a tool. The basic parameters are set as follows: population size ${N}_{sum}=100$, independent operation times 30, and dimension *dim* = *50*.

### Ablation experiment

In the ablation experiment, 28 benchmark functions are used to verify the effectiveness of the algorithm. These functions are selected from the CEC 2013 test suite^33^, where F1–F5 are unimodal functions, F6–F20 are basic multimode functions, and F21–F28 are combined functions, and the number of evaluations is controlled at the dimension multiplied by 10,000. The improvements used in the IIHHO algorithm were decomposed and combined to obtain a total of six variant algorithms, as shown in Table 1. Since the original HHO algorithm did not add parameters, all the variant algorithms did not set parameter initialization. By comparing IIHHO with HHO and six variant algorithms under the same constraints. In the experiment, the CEC 2013 function set is reset to the total test set to verify the algorithm performance, since the initial overall scale is set to 100 at the beginning of the experimental part, when the dimension of the problem is 50, the maximum number of function evaluations is 500,000. These algorithms run 30 times on each function, and take the Best value, Average value, Worst value and the Standard deviation(Std) value as evaluation indexes. In order to analyze the data, the average value is used to sort each algorithm. If the average values are equal, the standard deviation is considered in order to obtain a clear sequential relationship between the algorithms, and the experimental results are shown in Table 2.

**Table 1:** Settings for 6 variant algorithms.

Algorithms	Settings
IIHHO-I	*Combined with intermittent energy regulation strategy*
IIHHO-II	*Combined with attenuation vector factor*
IIHHO-III	*Combined with step size strategy based on Cardano formula*
IIHHO-IV	*Merged* IIHHO-I *and* IIHHO-II
IIHHO-V	*Merged* IIHHO-I *and* IIHHO-III
IIHHO-VI	*Merged* IIHHO-II *and* IIHHO-III

**Table 2:** Optimization results of each variant algorithm in 50 dimensions.

F	Index	IIHHO	IIHHO-I	IIHHO-II	IIHHO-III	IIHHO-IV	IIHHO-V	IIHHO-VI	HHO
F1	Best	**1.2026E−06**	1.0388E+01	3.1399E−02	5.6734E−01	4.7410E−06	5.7101E+00	3.2975E−02	1.4835E+01
	Aver	**2.5560E−06**	1.2818E+01	6.9141E−02	9.7839E−01	7.2028E−06	7.6441E+00	6.1136E−02	2.1504E+01
	Worst	**4.1952E−06**	1.5202E+01	1.0740E−01	1.4477E+00	1.1961E−05	9.5457E+00	9.4294E−02	3.1150E+01
	Std	**7.8475E−07**	1.4846E+00	1.9923E−02	1.9905E−01	1.7522E−06	8.5395E−01	1.6593E−02	3.4969E+00
	Rank	1	7	4	5	2	6	3	8
F2	Best	**1.3979E+06**	3.9233E+06	1.8904E+07	6.8661E+06	1.5155E+06	3.7321E+06	1.4676E+07	9.7980E+06
	Aver	3.1121E+06	6.7274E+06	3.5881E+07	1.6415E+07	**2.5440E+06**	6.9742E+06	3.1353E+07	1.7059E+07
	Worst	**5.6363E+06**	1.0858E+07	6.4883E+07	2.6027E+07	7.6491E+06	1.0191E+07	6.2739E+07	2.7204E+07
	Std	**9.7538E+05**	1.8817E+06	1.0393E+07	4.5854E+06	1.2163E+06	1.6376E+06	1.3000E+07	3.9989E+06
	Rank	2	3	8	5	1	4	7	6
F3	Best	**5.3678E+07**	1.1274E+08	3.1475E+09	4.6371E+08	9.8824E+07	1.9127E+08	3.2623E+09	4.0473E+08
	Aver	**5.6785E+08**	8.4682E+08	1.2273E+10	2.5271E+09	5.7426E+08	6.2123E+08	1.5875E+10	2.0439E+09
	Worst	2.6411E+09	4.0299E+09	2.9517E+10	8.9776E+09	2.1082E+09	**1.5901E+09**	5.1499E+10	5.8042E+09
	Std	5.5121E+08	8.5481E+08	5.9378E+09	1.8074E+09	5.1161E+08	**3.3301E+08**	9.7381E+09	1.4031E+09
	Rank	1	4	7	6	2	3	8	5
F4	Best	2.4121E+02	**1.3229E+02**	2.8021E+04	4.1481E+03	4.7851E+02	1.8204E+02	2.7501E+04	1.7948E+03
	Aver	6.9548E+02	**2.4079E+02**	3.7283E+04	7.3574E+03	2.4070E+03	3.6024E+02	3.7399E+04	3.9210E+03
	Worst	2.6605E+03	**3.3778E+02**	5.7557E+04	1.0833E+04	4.4586E+03	5.9628E+02	4.8077E+04	6.5407E+03
	Std	4.6837E+02	**5.1717E+01**	6.6353E+03	1.9343E+03	1.1144E+03	8.6731E+01	5.0269E+03	1.1300E+03
	Rank	3	1	7	6	4	2	8	5
F5	Best	**1.6534E−03**	2.7123E+00	1.5151E−01	4.7787E−01	3.3807E−03	1.8976E+00	1.6127E−01	4.5514E+00
	Aver	**2.0684E−03**	3.2729E+00	3.9402E−01	6.6994E−01	4.6442E−03	2.8147E+00	8.0732E−01	5.9451E+00
	Worst	**2.6776E−03**	3.9465E+00	1.0775E+01	9.5756E−01	5.9191E−03	3.3892E+00	4.4771E+00	7.5061E+00
	Std	**2.8307E−04**	3.0758E−01	2.3228E−01	1.3264E−01	6.6034E−04	3.4380E−01	1.0513E+00	6.4544E−01
	Rank	1	7	3	4	2	6	5	8
F6	Best	4.3451E+01	4.4190E+01	4.5081E+01	4.3744E+01	**2.9966E+01**	4.4141E+01	4.8315E+01	4.6922E+01
	Aver	6.8397E+01	8.4304E+01	1.2137E+02	9.1853E+01	**6.7733E+01**	8.7374E+01	1.4014E+02	9.6562E+01
	Worst	**1.0057E+02**	1.6311E+02	2.0395E+02	1.9699E+02	1.4558E+02	1.5661E+02	3.1503E+02	1.8052E+02
	Std	**2.5057E+01**	3.7841E+01	4.9256E+01	3.6929E+01	2.8499E+01	3.8660E+01	6.1535E+01	3.9453E+01
	Rank	2	3	7	5	1	4	8	6
F7	Best	1.1391E+02	1.2452E+02	1.3760E+02	1.2876E+02	**1.1312E+02**	1.4036E+02	1.3988E+02	1.5161E+02
	Aver	2.4829E+02	**2.0060E+02**	4.3473E+02	4.0013E+02	2.8730E+02	3.9808E+02	1.6331E+03	6.1899E+02
	Worst	1.3637E+03	**3.0660E+02**	2.0419E+03	1.9219E+03	1.3126E+03	5.1132E+03	8.9938E+03	6.9873E+03
	Std	2.2467E+02	**5.5279E+01**	4.2659E+02	3.6102E+02	2.6785E+02	8.9273E+02	2.5337E+03	1.2940E+03
	Rank	2	1	6	5	3	4	8	7
F8	Best	2.0951E+01	2.1008E+01	2.0997E+01	2.1009E+01	2.0989E+01	**2.0948E+01**	2.0955E+01	2.1047E+01
	Aver	2.1113E+01	2.1122E+01	2.1113E+01	2.1113E+01	2.1121E+01	2.1113E+01	**2.1108E+01**	2.1113E+01
	Worst	2.1190E+01	**2.1127E+01**	2.1168E+01	2.1171E+01	2.1197E+01	2.1182E+01	2.1155E+01	2.1183E+01
	Std	5.8216E−02	4.2213E−02	4.2650E−02	3.9174E−02	4.7598E−02	5.3414E−02	4.8263E−02	**3.5314E−02**
	Rank	3	8	4	5	7	2	1	6
F9	Best	**5.5637E+01**	5.5847E+01	5.9881E+01	5.7448E+01	5.6205E+01	5.7003E+01	6.3112E+01	5.8191E+01
	Aver	**6.4211E+01**	6.5287E+01	6.9914E+01	6.7671E+01	6.5604E+01	6.8993E+01	7.0553E+01	6.6320E+01
	Worst	**7.0925E+01**	7.2298E+01	7.5383E+01	7.6396E+01	7.5962E+01	7.4548E+01	7.5810E+01	7.4180E+01
	Std	4.1784E+00	4.3428E+00	3.2424E+00	4.5744E+00	5.4095E+00	4.4186E+00	**3.2398E+00**	4.0409E+00
	Rank	1	2	7	5	3	6	8	4
F10	Best	**2.5180E−02**	4.1133E+00	2.6596E+01	6.8335E+00	5.2269E−02	4.2022E+00	3.5742E+01	1.3581E+01
	Aver	1.9339E−01	6.2199E+00	5.3543E+01	1.2652E+01	**1.4718E−01**	5.8555E+00	6.7847E+01	2.2158E+01
	Worst	5.2996E−01	9.1569E+00	9.8702E+01	1.9681E+02	**3.4484E−01**	8.0963E+00	1.1373E+02	3.9245E+01
	Std	1.0045E−01	1.4744E+00	1.5952E+01	3.3560E+01	**6.7027E−02**	1.1335E+00	2.3127E+01	5.5769E+00
	Rank	2	4	7	5	1	3	8	6
F11	Best	**2.3879E+01**	1.3849E+02	3.9407E+02	2.2384E+02	6.6662E+01	2.1053E+02	4.2102E+02	3.1798E+02
	Aver	**3.4686E+01**	2.2585E+02	4.8733E+02	3.1502E+02	1.0649E+02	2.7196E+02	5.0711E+02	4.1388E+02
	Worst	**4.7758E+01**	2.9998E+02	5.5622E+02	3.8569E+02	1.3730E+02	3.3455E+02	6.4687E+02	5.5745E+02
	Std	**5.8960E+00**	3.5317E+01	4.1546E+01	3.8072E+01	1.8429E+01	3.0326E+01	5.6211E+01	5.4543E+01
	Rank	1	3	7	5	2	4	8	6
F12	Best	**5.7309E+02**	7.2378E+02	7.4955E+02	6.8970E+02	6.2781E+02	7.5291E+02	6.1001E+02	6.9973E+02
	Aver	**8.7495E+02**	9.4323E+02	9.0264E+02	9.3085E+02	8.7863E+02	9.2125E+02	8.7683E+02	9.8038E+02
	Worst	**1.0327E+03**	1.1134E+03	1.0546E+03	1.0735E+03	1.1223E+03	1.0596E+03	1.0417E+03	1.1615E+03
	Std	1.0584E+02	9.4090E+01	8.8306E+01	9.2748E+01	1.0402E+03	8.2420E+01	**9.6404E+01**	1.0010E+02
	Rank	1	7	4	6	3	5	2	8
F13	Best	8.5578E+02	9.3103E+02	9.1895E+02	8.5883E+02	**8.1923E+02**	8.4823E+02	9.3451E+02	8.7609E+02
	Aver	1.0335E+03	1.0957E+03	1.1019E+03	1.0708E+03	1.0915E+03	1.1077E+03	1.0983E+03	**1.0211E+03**
	Worst	**1.2149E+03**	1.2700E+03	1.3078E+03	1.2529E+03	1.2892E+03	1.2975E+03	1.3091E+03	1.2248E+03
	Std	9.6476E+01	9.3065E+01	1.1076E+02	1.0117E+02	1.1619E+02	1.1700E+02	9.1755E+01	**8.8077E+01**
	Rank	2	5	7	3	4	8	6	1
F14	Best	**1.9270E+02**	1.5701E+03	3.9198E+03	2.5388E+03	4.4331E+02	2.3035E+03	3.3094E+03	3.9099E+03
	Aver	**7.3278E+02**	3.7425E+03	5.8696E+03	4.3643E+03	1.3571E+03	3.8725E+03	5.5016E+03	6.3871E+03
	Worst	**2.1198E+03**	5.2582E+03	1.0571E+04	6.5236E+03	2.6615E+03	7.2112E+03	8.6120E+03	9.7101E+03
	Std	**5.0173E+02**	9.9961E+02	1.0992E+03	1.0242E+03	6.0676E+02	1.0486E+02	1.3729E+03	1.5135E+03
	Rank	1	3	7	5	2	4	6	8
F15	Best	**5.8752E+03**	7.7211E+03	7.4596E+03	7.2826E+03	7.2931E+03	6.6279E+03	7.7409E+03	7.7828E+03
	Aver	**7.8834E+03**	8.9320E+03	9.2908E+03	9.3140E+03	8.2983E+03	8.7601E+03	9.2411E+03	1.0147E+04
	Worst	**9.2689E+03**	1.0345E+04	1.1596E+04	1.2315E+04	9.5347E+03	1.0815E+04	1.1677E+04	1.2492E+04
	Std	9.1458E+02	7.3671E+02	1.0992E+03	1.0473E+03	**6.3189E+02**	9.7318E+02	1.0771E+03	1.1009E+03
	Rank	1	4	6	7	2	3	5	8
F16	Best	7.4103E−01	1.7353E+00	8.6752E−01	1.1139E+00	**4.9706E−01**	1.4028E+00	6.0744E−01	1.4441E+00
	Aver	1.5354E+00	2.5706E+00	1.9016E+00	2.2974E+00	**1.3895E+00**	2.1304E+00	2.0477E+00	2.4478E+00
	Worst	2.8618E+00	3.3618E+00	3.3727E+00	3.3110E+00	**2.6411E+00**	3.5532E+00	3.4017E+00	3.4268E+00
	Std	5.5573E−01	4.2464E−01	6.2077E−01	**5.5063E−01**	5.1081E−01	4.3028E−01	6.5181E−01	4.6412E−01
	Rank	2	8	3	6	1	5	4	7
F17	Best	**1.9370E+02**	4.9667E+02	8.2517E+02	9.4467E+02	5.3497E+02	7.9316E+02	7.9361E+02	9.8270E+02
	Aver	**2.5106E+02**	8.1862E+02	9.5548E+02	1.0559E+03	6.6593E+02	1.0769E+03	9.4142E+02	1.1621E+03
	Worst	**2.9596E+02**	1.2917E+03	1.0931E+02	1.1431E+03	8.5175E+02	1.2685E+03	1.1249E+03	1.3120E+03
	Std	**2.8561E+01**	1.6971E+02	6.9331E+01	5.6724E+01	9.5759E+01	1.1983E+02	8.2704E+01	7.0426E+01
	Rank	1	3	5	6	2	7	4	8
F18	Best	8.2170E+02	7.9712E+02	8.9162E+02	9.6887E+02	**7.3798E+02**	1.0303E+03	9.1338E+02	9.1227E+02
	Aver	1.0697E+03	1.0481E+03	1.0767E+03	1.1302E+03	**1.0153E+03**	1.2269E+03	1.1006E+03	1.1656E+03
	Worst	**1.1697E+03**	1.3198E+03	1.1835E+03	1.2429E+03	1.1848E+03	1.3852E+03	1.1907E+03	1.2608E+03
	Std	7.9219E+01	1.4084E+02	7.1085E+01	7.2272E+01	1.0959E+02	8.9876E+01	**6.9814E+01**	7.8308E+01
	Rank	3	2	4	6	1	8	5	7
F19	Best	**1.2222E+01**	2.8215E+01	7.9197E+01	5.4024E+01	1.6244E+01	3.5256E+01	8.5550E+01	5.8764E+01
	Aver	**1.9603E+01**	4.4568E+01	1.4323E+02	9.5999E+01	3.1694E+01	5.3527E+01	1.7270E+02	8.8669E+01
	Worst	**3.4330E+01**	6.2444E+01	2.8486E+02	1.5914E+02	5.0248E+01	7.6523E+01	2.4643E+02	1.3039E+02
	Std	**4.7558E+00**	9.0594E+00	3.7141E+01	2.4177E+01	8.7406E+00	1.0428E+01	3.8854E+01	2.1525E+01
	Rank	1	3	7	6	2	4	8	5
F20	Best	2.4500E+01	2.4008E+01	2.4046E+01	2.4067E+01	**2.3221E+01**	2.4019E+01	2.4500E+01	2.3855E+01
	Aver	2.4553E+01	2.4517E+01	2.4499E+01	**2.4480E+01**	2.4494E+01	2.4498E+01	2.4510E+01	2.4485E+01
	Worst	2.5000E+01	2.5000E+01	**2.4727E+01**	2.5000E+01	2.5000E+01	2.5000E+01	2.5000E+01	2.4996E+01
	Std	1.5164E−01	1.8019E−01	9.4731E−02	1.6605E−01	3.0134E−01	1.9026E−01	**5.7121E−03**	1.7513E−01
	Rank	8	7	5	1	3	4	6	2
F21	Best	**2.0004E+02**	8.3757E+02	8.3644E+02	2.2121E+02	2.0005E+02	2.5163E+02	2.0391E+02	8.3834E+02
	Aver	9.7718E+02	1.0119E+03	1.0270E+03	**9.1152E+02**	9.3907E+02	9.8146E+02	9.1620E+02	9.7535E+02
	Worst	**1.1223E+03**	1.1287E+03	1.1224E+03	1.1231E+03	1.1223E+03	1.1271E+03	1.1225E+03	1.1335E+03
	Std	2.0336E+02	1.4438E+02	**1.3705E+02**	1.8844E+02	1.9921E+02	1.9789E+02	2.7752E+02	1.4813E+02
	Rank	5	7	8	1	3	6	2	4
F22	Best	**7.7917E+02**	2.3047E+03	5.7814E+03	4.3020E+03	1.0978E+03	3.0289E+03	6.2513E+03	5.9930E+03
	Aver	**1.4132E+03**	4.0044E+03	9.2255E+03	6.6579E+03	2.0843E+03	4.4588E+03	8.5782E+03	8.0529E+03
	Worst	**2.5989E+03**	5.6073E+03	1.1768E+04	1.0113E+04	3.6214E+03	7.7803E+03	1.1143E+04	1.1356E+04
	Std	**5.4285E+02**	8.2321E+02	1.4497E+03	1.4607E+03	7.6039E+02	1.1111E+03	1.1090E+03	1.3394E+03
	Rank	1	3	8	5	2	4	7	6
F23	Best	**7.1143E+03**	7.8923E+03	8.5471E+03	9.9366E+03	7.2303E+03	9.3833E+03	7.9640E+03	1.0206E+04
	Aver	1.1332E+04	**1.1047E+04**	1.2192E+04	1.2106E+04	1.1055E+04	1.1739E+04	1.2188E+04	1.2473E+04
	Worst	1.4020E+04	1.3526E+04	1.4795E+04	1.3571E+04	**1.3017E+04**	1.3691E+04	1.4489E+04	1.4755E+04
	Std	1.3637E+03	1.2794E+03	1.2422E+03	**1.0472E+03**	1.3397E+03	1.2206E+03	1.5285E+03	1.1831E+03
	Rank	3	1	7	5	2	4	6	8
F24	Best	3.9531E+02	3.9722E+02	4.1438E+02	3.9519E+02	3.8804E+02	3.9621E+02	4.2014E+02	**3.6988E+02**
	Aver	4.2499E+02	4.2957E+02	4.4736E+02	4.2413E+02	4.2987E+02	4.3328E+02	4.6191E+02	**4.1753E+02**
	Worst	**4.5238E+02**	4.6316E+02	4.9157E+02	4.7574E+02	4.6605E+02	4.9063E+02	5.3890E+02	4.5805E+02
	Std	1.7149E+01	1.8602E+01	1.9453E+01	**1.7046E+01**	2.3255E+01	2.1740E+01	2.7423E+01	2.0608E+01
	Rank	3	4	7	2	5	6	8	1
F25	Best	4.0826E+02	**3.8526E+02**	4.0994E+02	4.0961E+02	4.1144E+02	3.8692E+02	4.1779E+02	4.0857E+02
	Aver	4.3815E+02	4.4545E+02	4.6301E+02	4.4241E+02	4.4886E+02	4.5158E+02	4.7364E+02	**4.3773E+02**
	Worst	**4.7136E+02**	4.8732E+02	5.1873E+02	4.8441E+02	5.1119E+02	5.2411E+02	5.5925E+02	4.7189E+02
	Std	1.7820E+01	2.2398E+01	**1.0812E+01**	2.0423E+01	2.4232E+01	2.9238E+01	4.5338E+01	1.5935E+01
	Rank	2	4	7	3	5	6	8	1
F26	Best	4.4818E+02	2.0107E+02	4.7347E+02	4.6257E+02	4.5862E+02	**2.0088E+02**	4.7059E+02	2.0167E+02
	Aver	4.8248E+02	4.6669E+02	4.9036E+02	4.8565E+02	4.8376E+02	4.7451E+02	4.9390E+02	**4.6002E+02**
	Worst	5.1156E+02	**4.9745E+02**	5.1873E+02	4.9984E+02	5.0375E+02	5.0638E+02	5.1644E+02	4.9789E+02
	Std	1.4061E+01	5.1331E+01	1.0811E+01	**1.0640E+01**	1.3126E+01	5.3608E+01	1.1006E+01	7.0709E+01
	Rank	4	2	7	6	5	3	8	1
F27	Best	1.9985E+03	**1.9901E+03**	2.1896E+03	1.9719E+03	2.0805E+03	1.8814E+03	2.1516E+03	2.0648E+03
	Aver	2.3133E+03	2.2732E+03	2.4312E+03	2.3074E+03	2.3702E+03	2.3974E+03	2.4055E+03	**2.2571E+03**
	Worst	2.6178E+03	2.5561E+03	2.7402E+03	2.6409E+03	2.7155E+03	2.9001E+03	2.8276E+03	**2.4725E+03**
	Std	1.4321E+02	1.4646E+02	1.4783E+02	1.7244E+02	1.6669E+02	1.6087E+02	1.5760E+02	**1.2114E+02**
	Rank	4	2	8	3	5	6	7	1
F28	Best	6.5876E+03	6.6504E+03	6.7182E+03	7.3415E+03	**4.0002E+02**	6.9616E+03	6.6070E+03	5.4885E+03
	Aver	8.1189E+03	8.2776E+03	8.4578E+03	8.3959E+03	**8.0991E+03**	8.5096E+03	8.6805E+03	8.1799E+03
	Worst	9.5328E+03	9.9598E+03	9.8424E+03	9.8467E+03	**9.2785E+03**	9.8916E+03	9.9971E+03	1.0193E+04
	Std	7.3038E+02	7.3933E+02	7.1223E+02	6.5139E+02	1.5877E+03	**6.4127E+02**	7.7146E+02	1.0953E+03
	Rank	2	4	6	5	1	7	8	3
Total rank		**2.2500**	4.0000	6.1786	4.7143	2.7857	4.7857	5.8571	5.2143

The minimum values under the same parameter comparison are in bold.

**Figure 3:**
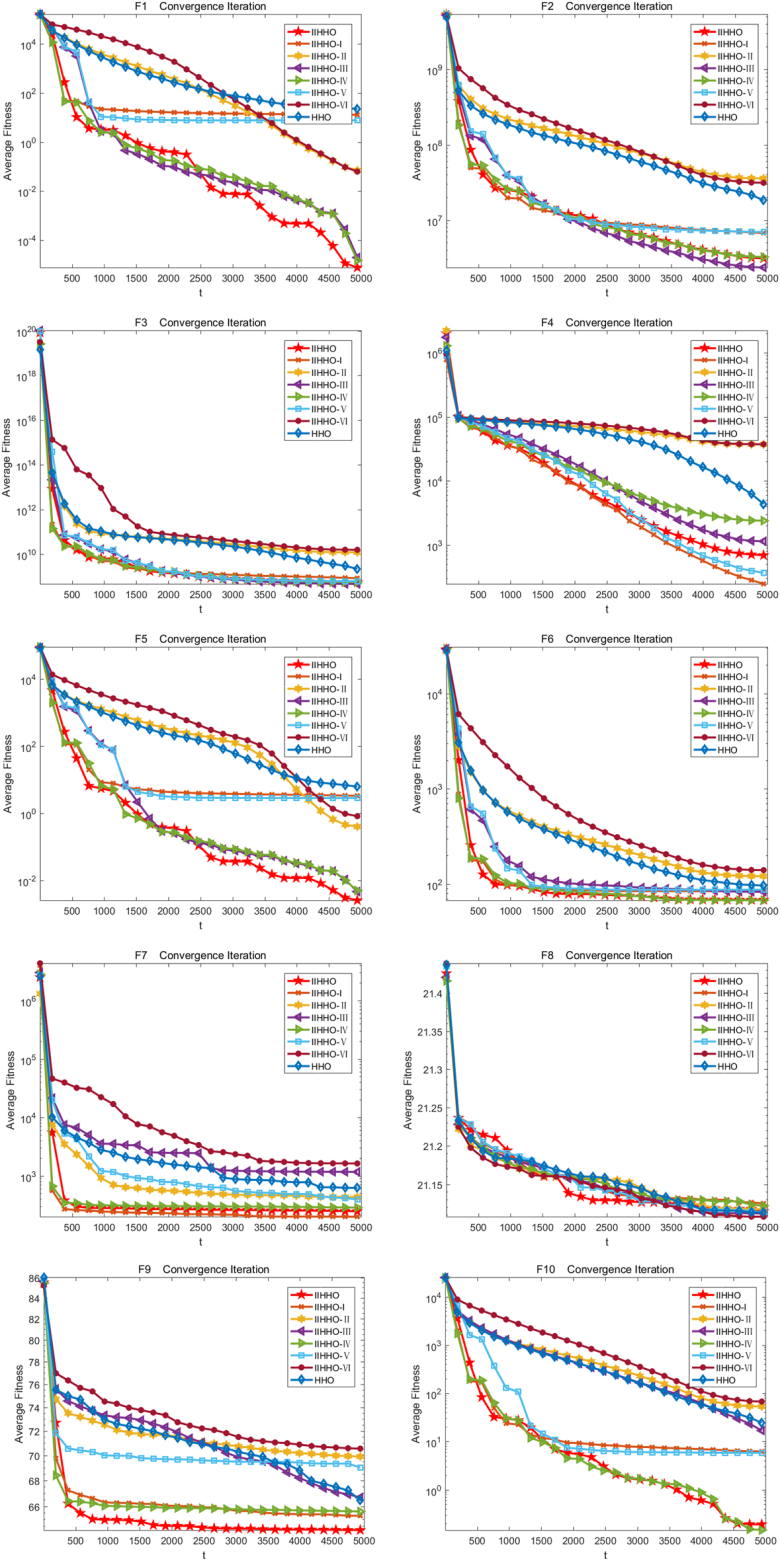

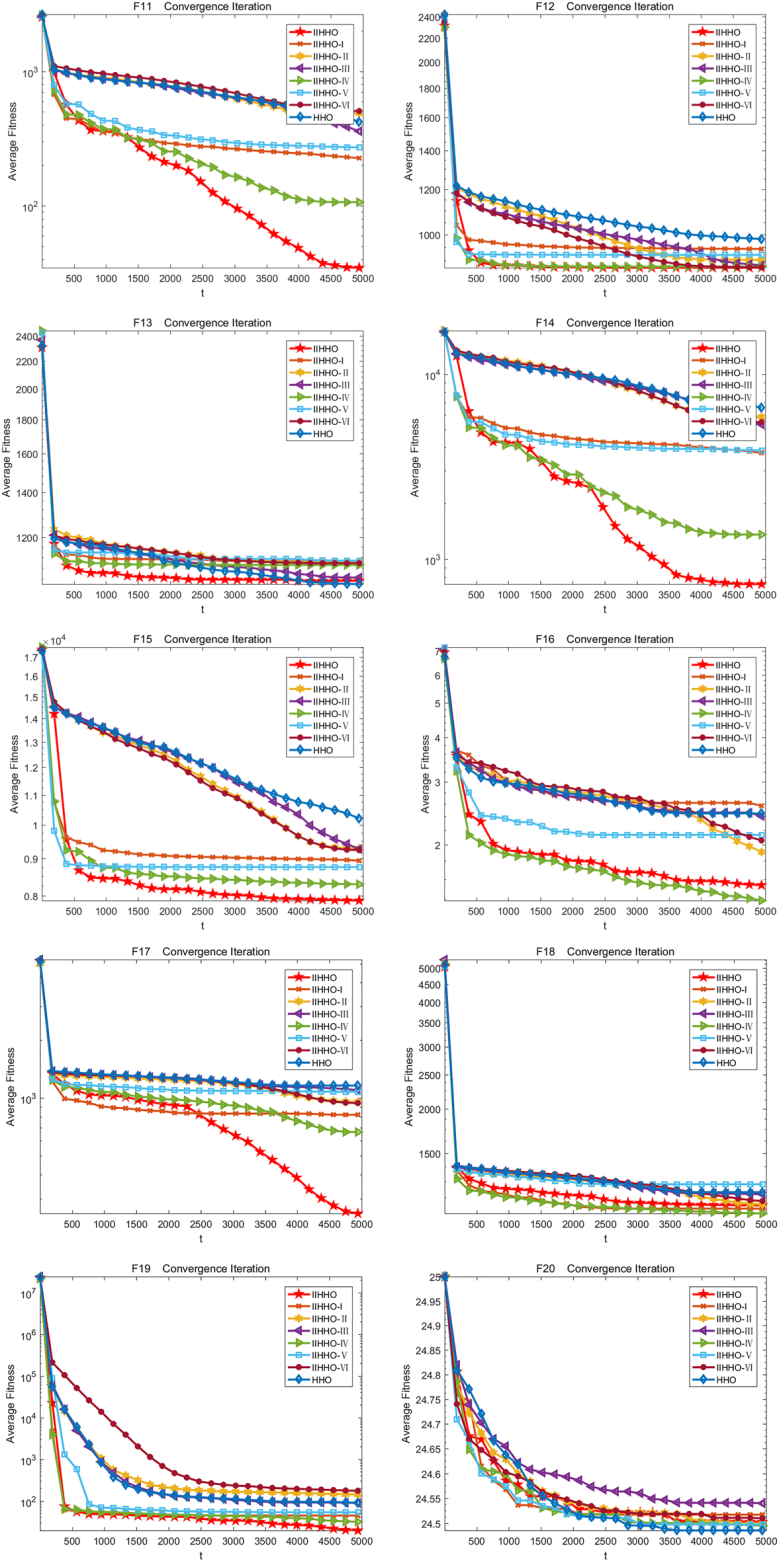

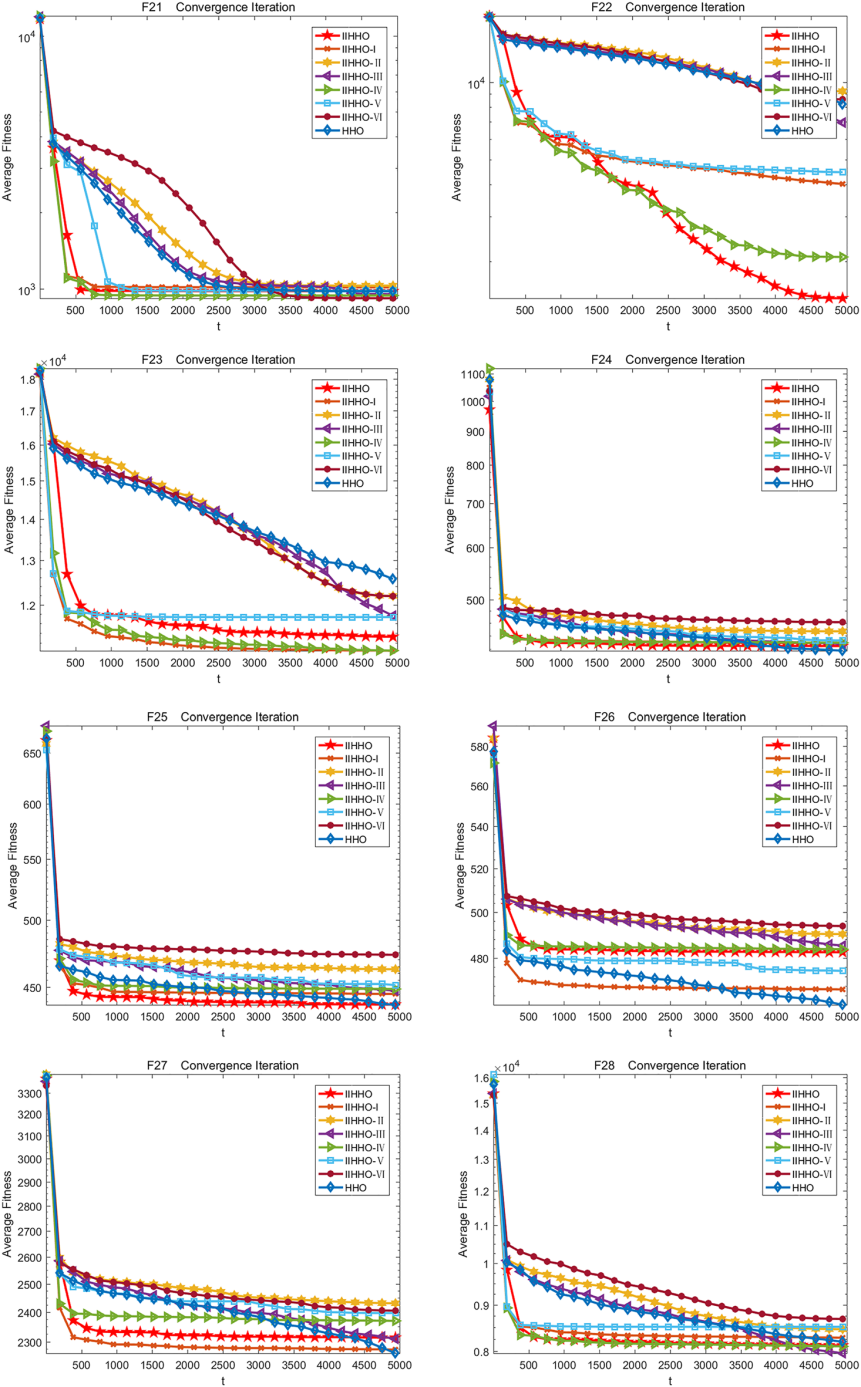
Iterative convergence graph of each improved algorithm.

In the experiment, the change of the average value of each algorithm in 5000 iterations is drawn to show the experimental effect of IIHHO more specifically. Finally, the iteration curves of eight algorithms are obtained, as shown in Fig. 3. As shown in Table 2, compared with the standard HHO algorithm, the proposed IIHHO algorithm is also superior to the other six IIHHO variant algorithms in the optimal value, worst value, average value and standard deviation of functions F1, F5, F11, F14, F17, F19 and F22. Similarly, in functions F2, F3, F9, F12, F15, F21, the numerical effect of IIHHO is also significantly dominant; Combined with ablation experiments, it is found that the improvement of energy and step size among the three improvement strategies can independently play a certain effect. Combined with the ranking of arithmetic results in Table 2, variant IIHHO-I and variant IIHHO-III algorithm are significantly better than HHO algorithm; Although the variant algorithm IIHHO-II with attenuation vector alone performs poorly, combined with the overall analysis, it is found that the optimization results of IIHHO in the 50 dimensional case are similar to IIHHO-IV, and it is much better than the original HHO algorithm in solving the problems of unimodal function and basic multi-mode function, which proves that adding attenuation vector is conducive to reducing the influence of Levy flight function and jumping out of the local optimum, and because IIHHO adds the step strategy combined with Cardano formula, The algorithm is often better than IIHHO-IV in solving the optimal value, which shows that the algorithm can maximize the excellent difference performance of the algorithm because each improvement strategy plays a corresponding role.

In order to further analyze whether there are differences between the existing improved algorithm and other algorithms, Wilcoxon test is used to verify the improvement effect of IIHHO algorithm. The principle of Wilcoxon test in this section is to compare each variable algorithm with the original HHO algorithm, and the final result is shown in the Context value in Table 3. Taking 0.05 as the bound, the Context value below 0.05 indicates that there is a significant difference between the two algorithms in the optimal value obtained by Wilcoxon test. First of all, after adding the intermittent energy regulator, the final IIHHO algorithm has greater differences than the original HHO algorithm in functions F1–F5 as shown in Table 3, and it is found that all values are excellent in combination with the experimental data in Table 2. Secondly, the two improved strategies of attenuation vector and Cardano formula can achieve the same effect, which proves the advantage of the improved strategy in improving the algorithm ability. Among unimodal functions and basic multimodal functions, IIHHO has greater differences, and according to the average index in Table 2, IIHHO can jump out of the local optimum and obtain a smaller average value.

**Table 3:** Wlicoxon test results of each variant algorithm.

F	Index	IIHHO	IIHHO-I	IIHHO-II	IIHHO-III	IIHHO-IV	IIHHO-V	IIHHO-VI
F1	Context	3.0199E−11(+)	4.0772E−11(+)	3.0199E−11(+)	3.0199E−11(+)	3.0199E−11(+)	3.0199E−11(+)	3.0199E−11(+)
F2	Context	3.0199E−11(+)	3.3384E−11(+)	2.3715E−10(−)	3.0199E−11(+)	3.0199E−11(+)	3.3383E−11(+)	5.4620E−06(−)
F3	Context	3.3520E−08(+)	2.8790E−06(+)	8.9934E−11(−)	1.1023E−08(−)	6.5277E−08(+)	2.6015E−08(+)	4.9752E−11(−)
F4	Context	4.9752E−11(+)	3.0199E−11(+)	3.0199E−11(−)	8.9934E−11(−)	2.7726E−05(+)	3.0199E−11(+)	3.0199E−11(−)
F5	Context	3.0199E−11(+)	3.0199E−11(+)	3.0199E−11(+)	3.0199E−11(+)	3.0199E−11(+)	3.0199E−11(+)	3.0199E−11(+)
F6	Context	1.1747E−04(+)	7.2446E−02(=)	4.3584E−02(−)	8.6844E−03(+)	7.1988E−05(+)	1.4532E−01(=)	5.0842E−03(−)
F7	Context	1.3703E−03(+)	7.6588E−05(+)	6.7350E−01(=)	1.2235E−01(=)	1.1228E−02(+)	4.0595E−02(+)	6.5486E−04(−)
F8	Context	4.1191E−01(=)	3.1830E−01(=)	5.7929E−01(=)	7.7311E−01(=)	1.8577E−01(=)	5.8945E−01(=)	9.3519E−01(=)
F9	Context	6.1452E−02(=)	3.4029E−01(=)	3.9881E−04(−)	7.0617E−01(=)	6.1001E−01(=)	8.3146E−03(−)	8.6634E−05(−)
F10	Context	3.0199E−11(+)	3.0199E−11(+)	9.9186E−11(−)	1.1567E−07(+)	3.0199E−11(+)	3.0199E−11(+)	4.0772E−11(−)
F11	Context	3.0199E−11(+)	3.0199E−11(+)	1.6062E−06(−)	1.9963E−05(+)	3.0199E−11(+)	3.6897E−11(+)	9.8329E−08(−)
F12	Context	1.5846E−04(+)	1.8090E−01(=)	1.9527E−03(+)	4.2175E−04(+)	2.0058E−04(+)	1.0763E−02(+)	1.1058E−04(+)
F13	Context	5.8945E−01(=)	4.4272E−03(−)	3.8481E−03(−)	2.3985E−01(=)	8.6844E−03(−)	2.8913E−03(−)	2.6509E−03(−)
F14	Context	3.0199E−11(+)	3.8249E−09(+)	1.3345E−01(=)	1.7666E−03(+)	3.0199E−11(+)	7.7725E−09(+)	3.2651E−02(+)
F15	Context	3.8249E−09(+)	2.1327E−05(+)	5.828E−03(+)	1.8575E−03(+)	2.1947E−08(+)	1.3367E−05(+)	2.3800E−03(+)
F16	Context	2.7829E−07(+)	3.6322E−01(=)	3.1821E−04(+)	7.5059E−01(=)	1.3111E−08(+)	2.4994E−03(+)	1.5638E−02(+)
F17	Context	3.0199E−11(+)	7.7725E−09(+)	1.4643E−10(+)	5.5611E−03(+)	3.0199E−11(+)	3.1830E−03(+)	2.6099E−10(+)
F18	Context	3.0939E−06(+)	8.1200E−04(+)	3.8349E−06(+)	6.7869E−02(=)	2.7829E−07(+)	7.2883E−03(−)	1.4067E−04(+)
F19	Context	3.0199E−11(+)	4.0772E−11(+)	3.4971E−09(−)	7.9585E−01(=)	3.0199E−11(+)	2.0338E−09(+)	4.1997E−10(−)
F20	Context	4.4074E−03(−)	7.6164E−01(=)	3.2495E−01(=)	8.9983E−01(=)	2.8964E−01(=)	9.1156E−01(=)	5.1027E−01(=)
F21	Context	8.6844E−03(−)	1.8368E−02(−)	5.5546E−02(=)	8.5641E−04(+)	3.5638E−04(+)	8.6844E−03(−)	1.9527E−03(+)
F22	Context	3.0199E−11(+)	3.0199E−11(+)	1.1738E−03(−)	4.4592E−04(+)	3.0199E−11(+)	2.1544E−10(+)	7.0127E−02(=)
F23	Context	2.6243E−03(+)	1.0407E−04(+)	5.8945E−01(=)	4.8413E−02(+)	2.2539E−04(+)	4.0595E−02(+)	6.3088E−01(=)
F24	Context	1.5367E−01(=)	3.3874E−02(−)	2.0023E−06(−)	6.7867E−02(=)	6.3532E−02(=)	1.1228E−02(−)	2.8314E−08(−)
F25	Context	9.3519E−01(=)	9.0490E−02(=)	1.5969E−03(−)	1.9073E−01(=)	6.3532E−02(=)	3.7782E−02(=)	2.6806E−04(=)
F26	Context	6.3533E−02(=)	5.5923E−01(=)	2.7726E−05(−)	7.2884E−03(−)	2.0681E−02(−)	2.2360E−02(−)	3.2555E−07(−)
F27	Context	1.1882E−01(=)	6.4142E−01(=)	3.1573E−05(−)	2.7071E−01(=)	6.6689E−03(−)	9.2113E−05(−)	3.3679E−04(−)
F28	Context	5.8945E−01(=)	9.5873E−01(=)	3.4783E−01(=)	4.4642E−01(=)	8.5338E−01(=)	2.9727E−01(=)	7.2446E−02(=)
+ / = /-		18/8/2	15/10/3	7/7/14	13/12/3	19/6/3	16/5/7	9/6/13

### Performance comparison between IIHHO and improved algorithms

After determining the improvement strategy adopted, this section uses IIHHO and the selected seven improved algorithms, LMHHO^34^, ISSA^35^, DPSO^36^, LHHO^37^, HHO_JOS^38^, CDO^39^ and SSO^40^ conducted data comparison and analysis, and the corresponding parameter settings are shown in Table 4. All algorithms are still tested in CEC 2013, and the maximum number of function evaluations is 500,000. The experimental results are shown in Table 5. The results of Wilcoxon test are listed in Table 6.

**Table 4:** Parameter settings for each improved algorithm.

Algorithms	Parameters
LMHHO	$MemoryLimit=10, \;\; gBestCollCount=0$
ISSA	$P\_percent=0.2$
DPSO	$c1init=2.5$*;*$c1fin=0.5$*;*$c2init=0.5$*;*$c2init=2.5$
LHHO	–
HHO_JOS	$BFid=1,\;\;Jr=0.25$
CDO	–
SSO	$step=1$

**Table 5:** Optimization results of each improved algorithm in 50 dimensions.

F	Index	LMHHO	ISSA	DPSO	LHHO	HHO_JOS	CDO	SSO	IIHHO
F1	Best	1.3402E+04	9.2300E−02	1.0348E+04	1.4507E+01	7.5312E−04	4.6040E+04	4.1628E+04	**1.2026E−06**
	Aver	2.4963E+04	2.7080E+04	2.2763E+04	2.3219E+01	1.9573E−03	4.6508E+04	4.7095E+04	**2.5560E−06**
	Worst	3.5193E+04	6.8192E+04	3.5495E+04	3.1701E+01	1.9556E−03	4.6883E+04	5.4488E+04	**4.1952E−06**
	Std	5.0162E+03	2.8314E+04	6.5949E+03	4.3795E+00	8.1096E−04	1.9732E+02	3.5180E+03	**7.8475E−07**
	Rank	5	6	4	3	2	7	8	1
F2	Best	1.7293E+08	9.5752E+06	9.1875E+06	1.7777E+07	**2.5803E+05**	7.0532E+08	2.6935E+08	1.3979E+06
	Aver	3.8245E+08	2.2169E+08	7.7304E+07	2.8026E+07	5.4186E+06	7.7874E+08	4.1753E+08	**3.1121E+06**
	Worst	7.4317E+08	1.1018E+09	5.4306E+08	4.0007E+07	8.3123E+06	8.9097E+08	5.6559E+08	**5.6363E+06**
	Std	1.4642E+08	3.3452E+08	1.0316E+08	5.3224E+06	1.7414E+06	3.8019E+07	7.0428E+07	**9.7538E+05**
	Rank	6	5	4	3	2	8	7	1
F3	Best	8.4811E+10	7.7921E+08	2.3672E+08	9.8355E+08	1.8675E+08	6.1988E+10	8.7760E+10	**5.3678E+07**
	Aver	4.2102E+13	4.8544E+12	4.7841E+10	3.5447E+09	1.0811E+09	7.2732E+10	2.8932E+11	**5.6785E+08**
	Worst	7.0517E+13	4.5138E+13	1.8684E+11	1.1179E+10	3.6426E+09	8.8930E+10	2.3257E+12	**2.6411E+09**
	Std	1.3414E+13	1.2006E+12	5.5817E+10	2.3992E+09	8.4573E+08	6.4252E+09	4.2756E+11	**5.5121E+08**
	Rank	8	7	4	3	2	5	6	1
F4	Best	7.9878E+04	3.4555E+04	9.4789E+03	5.8627E+03	1.3619E+03	5.7170E+04	6.5101E+04	**2.4121E+02**
	Aver	8.6762E+04	6.0590E+04	2.3084E+04	9.1578E+03	4.2792E+03	6.1765E+04	7.4630E+04	**6.9548E+02**
	Worst	9.8190E+04	8.3573E+04	3.3842E+04	1.7086E+04	4.3658E+03	6.8452E+04	8.7345E+04	**2.6605E+03**
	Std	4.3494E+03	1.5719E+04	6.9414E+03	2.5005E+03	1.6123E+03	2.5862E+03	4.8177E+03	**4.6837E+02**
	Rank	8	5	4	3	2	6	7	1
F5	Best	1.9781E+03	1.9723E−01	7.1847E+02	5.5621E+00	5.1411E−02	1.0667E+04	3.3903E+03	**1.6534E−03**
	Aver	3.5517E+03	4.0030E+03	2.4041E+03	7.1018E+00	6.0020E−02	3.9023E+04	5.8816E+03	**2.0684E−03**
	Worst	5.3996E+03	9.9078E+03	5.5397E+03	8.9259E+00	7.0977E−02	5.1158E+04	8.9797E+03	**2.6776E−03**
	Std	9.4709E+02	4.4040E+03	1.0986E+03	8.7790E−01	5.0170E−03	1.8853E+04	1.2613E+03	**2.8307E−04**
	Rank	5	6	4	3	2	8	7	1
F6	Best	8.7895E+02	4.5722E+01	2.7506E+02	4.4704E+01	4.3458E+01	3.2382E+03	2.6141E+03	**4.3451E+01**
	Aver	1.7305E+03	2.2914E+03	1.4310E+03	1.0117E+02	8.3649E+01	3.3333E+03	3.3147E+03	**6.8397E+01**
	Worst	4.6325E+03	6.7171E+03	2.7431E+03	2.0578E+02	1.4896E+02	3.4142E+03	4.0700E+03	**1.0057E+02**
	Std	6.5967E+02	2.5629E+03	7.3425E+02	5.1161E+01	2.9648E+01	4.1128E+01	4.2357E+02	**2.5057E+01**
	Rank	5	6	4	3	2	8	7	1
F7	Best	1.9767E+02	**9.7201E+01**	9.1139E+01	1.0129E+02	1.1316E+02	1.3600E+02	2.1005E+02	1.1391E+02
	Aver	9.0140E+03	4.2598E+02	2.0426E+02	2.6219E+02	2.3937E+02	**1.6446E+02**	2.9095E+02	2.4829E+02
	Worst	9.4471E+04	1.2829E+03	5.2865E+02	1.0857E+03	7.3459E+02	**1.8486E+02**	4.7760E+02	1.3637E+03
	Std	2.3694E+04	3.8622E+02	9.3440E+01	1.8845E+02	1.2767E+02	**1.0152E+01**	6.0321E+01	2.2467E+02
	Rank	8	7	2	5	3	1	6	4
F8	Best	2.1007E+01	2.0960E+01	2.1028E+01	2.1017E+01	**2.0934E+01**	2.1084E+01	2.0997E+01	2.0951E+01
	Aver	2.1124E+01	2.1132E+01	2.1114E+01	2.1107E+01	**2.1100E+01**	2.1132E+01	2.1130E+01	2.1113E+01
	Worst	2.1182E+01	2.1201E+01	2.1175E+01	2.1200E+01	**2.1175E+01**	2.1180E+01	2.1179E+01	2.1190E+01
	Std	4.3063E−02	4.7965E−02	3.6660E−02	4.6345E−02	6.1825E−02	**2.6722E−02**	3.8838E−02	5.8216E−02
	Rank	5	8	4	2	1	7	6	3
F9	Best	6.7038E+01	5.2781E+01	**4.4878E+01**	5.7044E+01	5.7665E+01	5.8275E+01	6.7295E+01	5.5637E+01
	Aver	7.3184E+01	6.6978E+01	**5.9996E+01**	6.5322E+01	6.4922E+01	6.4791E+01	7.2726E+01	6.4211E+01
	Worst	7.9762E+01	7.6741E+01	**6.9101E+01**	7.2965E+01	**7**.1790E+01	7.2966E+01	7.5328E+01	7.0925E+01
	Std	3.4100E+00	5.6652E+00	5.9166E+00	3.7515E+00	3.6022E+00	3.4972E+00	**1.7263E+00**	4.1784E+00
	Rank	8	6	1	5	4	3	7	2
F10	Best	2.3870E+03	7.4836E+00	6.1986E+02	3.2944E+01	1.2693E+00	6.8391E+03	3.6248E+03	**2.5180E−02**
	Aver	3.6379E+03	1.9211E+03	2.3064E+03	4.8341E+01	1.6542E+00	6.9840E+03	4.7704E+03	**1.9339E−01**
	Worst	5.7994E+03	1.0612E+04	6.0406E+03	8.1595E+01	2.3750E+00	7.1372E+03	6.1226E+03	**5.2996E−01**
	Std	6.6681E+02	4.2186E+03	1.0639E+03	1.1041E+01	2.6326E−01	7.6127E+01	7.5931E+02	**1.0045E−01**
	Rank	6	4	5	3	2	8	7	1
F11	Best	7.4307E+02	3.9032E+02	3.7539E+02	3.6120E+02	1.4432E+02	9.7250E+02	7.9098E+02	**2.3879E+01**
	Aver	8.9560E+02	7.7517E+02	4.5973E+02	4.8003E+02	2.0567E+02	1.0242E+03	8.7168E+02	**3.4686E+01**
	Worst	1.0298E+03	1.0661E+03	5.4890E+02	6.4228E+02	2.7264E+02	1.0887E+03	9.6510E+02	**4.7758E+01**
	Std	7.5808E+01	2.4016E+02	3.9087E+01	6.4883E+01	3.5511E+01	1.0152E+01	3.7996E+01	**5.8960E+00**
	Rank	7	5	3	4	2	8	6	1
F12	Best	8.4809E+02	6.1538E+02	**4.1412E+02**	6.7476E+02	6.5866E+02	8.2888E+02	8.2402E+02	5.7309E+02
	Aver	1.1421E+03	9.4280E+02	**5.4620E+02**	8.9829E+02	8.0755E+02	8.8187E+02	8.8978E+02	8.7495E+02
	Worst	1.2547E+03	1.2976E+03	**6.9441E+02**	1.1442E+03	1.0137E+03	9.2226E+02	9.5026E+02	1.0327E+03
	Std	8.4584E+01	1.6838E+02	6.0624E+01	1.3533E+02	1.0818E+02	**2.5632E+01**	3.4114E+01	1.0584E+02
	Rank	8	7	1	6	2	4	5	3
F13	Best	1.0703E+03	6.2168E+02	**5.9400E+02**	7.2477E+02	7.2780E+02	8.2469E+02	7.7070E+02	8.5578E+02
	Aver	1.2114E+03	9.4280E+02	**7.3572E+02**	9.8282E+02	9.2990E+02	8.6625E+02	8.8639E+02	1.0335E+03
	Worst	1.3256E+03	1.2976E+03	**8.5216E+02**	1.2162E+03	1.1366E+03	9.1121E+02	9.9372E+02	1.2149E+03
	Std	6.6320E+01	1.6839E+02	7.0603E+01	1.2831E+02	1.0818E+02	**1.8688E+01**	5.0189E+01	9.6476E+01
	Rank	8	5	1	6	4	2	3	7
F14	Best	8.6950E+03	5.7386E+03	4.7342E+03	4.2500E+03	1.2832E+03	1.1229E+04	1.3194E+04	**1.9270E+02**
	Aver	1.0733E+04	1.0125E+04	5.9007E+03	6.6704E+03	2.3854E+03	1.2077E+04	1.4481E+04	**7.3278E+02**
	Worst	1.2917E+04	1.4668E+04	7.9709E+03	8.5567E+03	5.4732E+03	1.2970E+04	1.5404E+04	**2.1198E+03**
	Std	1.1686E+03	3.1755E+03	6.9230E+02	1.0248E+03	8.7583E+02	4.1838E+03	5.0542E+02	**5.0173E+02**
	Rank	6	5	3	4	2	7	8	1
F15	Best	1.2101E+04	7.6985E+03	**5.4789E+03**	8.2960E+03	7.1201E+03	1.3454E+04	1.2045E+04	5.8752E+03
	Aver	1.3754E+04	1.0650E+04	**7.7658E+03**	1.0442E+04	8.8021E+03	1.4160E+04	1.3989E+04	7.8834E+03
	Worst	1.5128E+04	1.4462E+04	1.0356E+04	1.2326E+04	1.1153E+04	1.4834E+04	1.4803E+04	**9.2689E+03**
	Std	6.4888E+02	2.3316E+03	1.0282E+03	8.7980E+02	9.1970E+02	**3.4812E+02**	6.2554E+02	9.1458E+02
	Rank	6	5	1	4	3	8	7	2
F16	Best	1.4423E+00	1.0634E+00	1.4555E+00	1.6054E+00	7.7311E−01	2.1792E+00	2.3923E+00	**7.4103E−01**
	Aver	2.7680E+00	2.2155E+00	2.6409E+00	2.3486E+00	1.9014E+00	3.2354E+00	3.2707E+00	**1.5354E+00**
	Worst	3.7606E+00	3.5075E+00	3.4454E+00	3.4050E+00	**2.6619E+00**	3.9754E+00	3.7312E+00	2.8618E+00
	Std	5.9262E−01	6.7582E−01	5.3072E−01	4.0107E−01	4.6392E−01	4.1149E−01	**2.9217E−01**	5.5573E−01
	Rank	6	3	5	4	2	7	8	1
F17	Best	1.1780E+03	7.4139E+02	4.7322E+02	9.5386E+02	5.9805E+02	9.6760E+02	1.0058E+03	**1.9370E+02**
	Aver	1.2741E+03	1.1826E+03	6.1916E+02	1.0985E+03	7.8727E+02	1.0286E+03	1.1131E+03	**2.5106E+02**
	Worst	1.3806E+03	1.3919E+03	7.0876E+02	1.2298E+03	9.8927E+02	1.0699E+03	1.2673E+03	**2.9596E+02**
	Std	4.3594E+01	1.4138E+02	5.9955E+01	7.0390E+01	9.1009E+01	**2.5501E+01**	6.2030E+01	2.8561E+01
	Rank	8	7	2	5	3	4	6	1
F18	Best	1.2485E+03	9.0989E+02	**4.3242E+02**	8.5231E+02	6.7040E+02	9.7395E+02	9.2784E+02	8.2170E+02
	Aver	1.2969E+03	1.1359E+03	**6.2574E+02**	1.1014E+03	9.6060E+02	1.0264E+03	1.0969E+03	1.0697E+03
	Worst	1.3714E+03	1.3619E+03	**8.1810E+02**	1.2521E+03	1.1714E+03	1.0943E+03	1.2217E+03	**1**.1697E+03
	Std	3.1076E+01	1.1448E+02	8.9131E+01	9.8754E+03	1.1724E+02	**2.4763E+01**	6.9741E+01	7.9219E+01
	Rank	8	7	1	6	2	3	5	4
F19	Best	6.6371E+03	3.4227E+01	3.9976E+01	5.6735E+01	6.3754E+01	1.3464E+06	4.0353E+04	**1.2222E+01**
	Aver	2.8124E+04	5.2276E+04	9.6242E+03	9.0304E+01	1.2050E+02	1.3543E+06	1.2423E+05	**1.9603E+01**
	Worst	6.8304E+04	4.3568E+05	4.1047E+04	1.4740E+02	2.5907E+02	1.3656E+06	3.1754E+05	**3.4330E+01**
	Std	1.5251E+04	1.0351E+05	1.0969E+04	2.1708E+01	4.2802E+01	5.1600E+03	6.2590E+04	**4.7558E+00**
	Rank	5	6	4	2	3	8	7	1
F20	Best	2.4502E+01	**2.1661E+01**	2.2576E+01	2.2914E+01	2.3514E+01	2.3959E+01	2.3650E+01	2.4500E+01
	Aver	2.4604E+01	2.4578E+01	**2.3734E+01**	2.4420E+01	2.4384E+01	2.4419E+01	2.4386E+01	2.4553E+01
	Worst	2.5000E+01	2.4579E+01	**2.4510E+01**	**2.4510E+01**	**2.4510E+01**	2.4678E+01	2.4679E+01	2.5000E+01
	Std	**1.4203E−01**	5.4976E−01	5.5642E−01	3.0993E−01	2.5446E−01	1.7681E−01	2.4406E−01	1.5164E−01
	Rank	8	7	1	5	2	4	3	6
F21	Best	3.2845E+03	8.3651E+02	7.8351E+02	2.8258E+02	8.3644E+02	3.8337E+03	3.9193E+03	**2.0004E+02**
	Aver	3.5393E+03	2.8347E+03	2.9891E+03	**9.4965E+02**	9.9841E+02	3.8461E+03	4.0181E+03	9.7718E+02
	Worst	3.8066E+03	4.3084E+03	3.8303E+03	1.1369E+03	1.1224E+03	3.8571E+03	4.1639E+03	**1.1223E+03**
	Std	1.2205E+02	1.5278E+03	7.7062E+02	2.3064E+02	1.4405E+02	**5.8365E+00**	7.3254E+01	2.0336E+02
	Rank	6	4	5	1	3	7	8	2
F22	Best	1.3108E+04	6.2121E+03	7.3342E+03	6.8297E+03	2.5282E+03	1.2890E+04	1.5112E+04	**7.7917E+02**
	Aver	1.4217E+04	1.2000E+04	9.1421E+03	8.3557E+03	4.1150E+03	1.3783E+04	1.6139E+04	**1.4132E+03**
	Worst	1.5164E+04	1.6460E+04	1.1070E+04	1.1358E+04	8.9964E+03	1.4560E+04	1.7074E+04	**2.5989E+03**
	Std	5.4564E+02	3.5325E+03	1.0335E+03	1.0696E+03	1.1996E+03	**4.1892E+02**	5.2314E+02	5.4285E+02
	Rank	7	5	4	3	2	6	8	1
F23	Best	1.4504E+04	9.1700E+03	8.2956E+03	9.7381E+03	1.0179E+04	1.3699E+04	1.5105E+04	**7.1143E+03**
	Aver	1.5625E+04	1.2394E+04	**1.0705E+04**	1.2286E+04	1.2231E+04	1.4980E+04	1.6139E+04	1.1332E+04
	Worst	1.6879E+04	1.6025E+04	**1.3796E+04**	1.4108E+04	1.3931E+04	1.6177E+04	1.7074E+04	1.4020E+04
	Std	6.3854E+02	2.1724E+03	1.1277E+03	1.2203E+03	1.1330E+03	**4.7765E+02**	5.2314E+02	1.3637E+03
	Rank	7	5	1	4	3	6	8	2
F24	Best	4.2162E+02	**3.5079E+02**	3.6096E+02	3.8319E+02	4.0023E+02	4.0699E+02	4.4674E+02	3.9531E+02
	Aver	5.1884E+02	4.1614E+02	**4.0923E+02**	4.2631E+02	4.2572E+02	4.2092E+02	4.7988E+02	4.2499E+02
	Worst	6.4806E+02	5.1056E+02	4.6917E+02	4.6144E+02	4.5948E+02	**4.4765E+02**	5.1500E+02	4.5238E+02
	Std	5.5372E+01	4.3798E+01	2.5995E+02	1.6619E+01	1.5875E+01	**1.0406E+01**	1.8312E+01	1.7149E+01
	Rank	8	2	1	6	5	3	7	4
F25	Best	4.3035E+02	**3.6856E+02**	4.4124E+02	4.1679E+02	4.0840E+02	4.6475E+02	4.9808E+02	4.0826E+02
	Aver	5.3633E+02	**4.2249E+02**	5.3271E+02	4.4794E+02	4.6989E+02	4.9032E+02	5.3428E+02	4.3815E+02
	Worst	5.6352E+02	4.8960E+02	5.9316E+02	5.0670E+02	5.2881E+02	5.1002E+02	5.6579E+02	**4.7136E+02**
	Std	4.4025E+01	2.9375E+01	3.4700E+01	1.8652E+01	3.1589E+01	**1.0422E+01**	1.6053E+01	1.7820E+01
	Rank	8	1	6	3	4	5	7	2
F26	Best	2.9979E+02	**2.0011E+02**	2.0138E+02	2.0168E+02	2.0044E+02	2.1580E+02	2.3183E+02	4.4818E+02
	Aver	4.9568E+02	**3.3262E+02**	4.4457E+02	4.6054E+02	3.5070E+02	4.6500E+02	4.2175E+02	4.8248E+02
	Worst	5.2389E+02	**4.8702E+02**	4.9844E+02	5.0670E+02	4.9709E+02	4.8829E+02	5.0740E+02	5.1156E+02
	Std	3.8653E+01	1.1550E+02	6.7413E+01	7.1429E+01	1.4099E+02	4.7888E+01	1.0923E+02	**1.4061E+01**
	Rank	8	1	4	5	2	6	3	7
F27	Best	2.2128E+03	1.8811E+03	**1.8183E+03**	2.0337E+03	1.9468E+03	1.9439E+03	2.2668E+03	1.9985E+03
	Aver	2.5861E+03	**2.1427E+03**	2.1893E+03	2.2463E+03	2.2680E+03	2.2193E+03	2.4606E+03	2.3133E+03
	Worst	2.9504E+03	2.7079E+03	2.5988E+03	**2.4228E+03**	2.4986E+03	2.4768E+03	2.6962E+03	2.6178E+03
	Std	2.0060E+02	2.1417E+02	1.9350E+02	1.1404E+02	1.6537E+02	1.2258E+02	**9.8553E+01**	1.4321E+02
	Rank	8	1	2	4	5	3	7	6
F28	Best	7.7076E+03	**4.0331E+02**	5.4885E+03	5.7890E+03	3.7366E+03	7.2111E+03	6.0924E+03	6.5876E+03
	Aver	9.7656E+03	**4.0051E+03**	6.4576E+03	7.7309E+03	6.9788E+03	7.3940E+03	6.8927E+03	8.1189E+03
	Worst	1.1220E+04	9.4392E+03	**7.2883E+03**	9.4423E+03	8.8623E+03	7.6822E+03	7.8265E+03	9.5328E+03
	Std	9.4420E+02	3.3170E+03	5.0986E+02	8.9894E+02	1.1302E+03	**1.1691E+02**	3.8978E+02	7.3038E+02
	Rank	8	1	2	6	4	5	3	7
Total rank		6.9286	4.8929	2.9643	3.9643	2.6786	5.6071	6.3214	**2.6429**

The minimum values under the same parameter comparison are in bold.

**Table 6:** Wlicoxon test results of each improved algorithm.

F	Index	LMHHO	ISSA	DPSO	LHHO	HHO_JOS	CDO	SSO
F1	Contest	3.0199E−11(+)	3.0199E−11(+)	3.0199E−11(+)	3.0199E−11(+)	3.0199E−11(+)	3.0199E−11(+)	3.0199E−11(+)
F2	Contest	3.0199E−11(+)	3.0199E−11(+)	3.0199E−11(+)	3.0199E−11(+)	1.1937E−06(+)	3.0199E−11(+)	3.0199E−11(+)
F3	Contest	3.0199E−11(+)	1.6132E−10(+)	1.3111E−08(+)	1.7769E−10(+)	3.3679E−04(+)	3.0199E−11(+)	3.0199E−11(+)
F4	Contest	3.0199E−11(+)	3.0199E−11(+)	3.0199E−11(+)	3.0199E−11(+)	5.8587E−06(+)	3.0199E−11(+)	3.0199E−11(+)
F5	Contest	3.0199E−11(+)	3.0199E−11(+)	3.0199E−11(+)	3.0199E−11(+)	3.0199E−11(+)	3.0199E−11(+)	3.0199E−11(+)
F6	Contest	3.0199E−11(+)	6.5183E−09(+)	3.0199E−11(+)	2.2658E−03(+)	1.9112E−02(+)	3.0199E−11(+)	3.0199E−11(+)
F7	Contest	1.7290E−06(+)	9.9258E−02(=)	1.2477E−04(−)	3.8481E−03(+)	1.4423E−03(−)	2.3768E−07(−)	2.9727E−01(=)
F8	Contest	3.2651E−02(+)	6.6689E−03(+)	3.3285E−01(=)	8.6499E−01(=)	4.5530E−01(=)	8.6844E−03(+)	6.6689E−03(+)
F9	Contest	6.3772E−03(+)	1.6798E−03(+)	2.0337E−09(−)	2.1540E−06(+)	4.4440E−07(+)	5.4620E−06(+)	4.8560E−03(+)
F10	Contest	3.0199E−11(+)	3.0199E−11(+)	3.0199E−11(+)	3.0199E−11(+)	3.0199E−11(+)	3.0199E−11(+)	3.0199E−11(+)
F11	Contest	3.0199E−11(+)	3.0199E−11(+)	3.0199E−11(+)	3.0199E−11(+)	4.5043E−11(+)	3.0199E−11(+)	3.0199E−11(+)
F12	Contest	1.6947E−09(+)	3.0199E−11(+)	3.0199E−11(−)	7.1719E−01(+)	8.5641E−04(−)	2.8378E−01(=)	6.1001E−01(=)
F13	Contest	1.0035E−03(+)	6.5486E−04(−)	3.0199E−11(−)	3.1573E−05(−)	3.3520E−08(−)	3.0199E−11(−)	3.3384E−11(−)
F14	Contest	3.0199E−11(+)	1.3367E−05(+)	3.0199E−11(+)	3.6897E−11(+)	7.6171E−03(+)	3.0199E−11(+)	3.0199E−11(+)
F15	Contest	3.0199E−11(+)	3.0199E−11(+)	2.1702E−01(=)	7.3803E−10(+)	1.3272E−02(+)	3.0199E−11(+)	3.0199E−11(+)
F16	Contest	1.1023E−08(+)	3.8349E−06(+)	8.4848E−09(+)	9.8329E−08(+)	3.0339E−03(+)	4.9752E−11(+)	3.3384E−11(+)
F17	Contest	3.0199E−11(+)	2.6806E−04(+)	3.5137E−02(+)	3.0199E−11(+)	4.7445E−06(+)	3.0199E−11(+)	3.0199E−11(+)
F18	Contest	3.0199E−11(+)	5.4941E−11(+)	3.0199E−11(−)	2.5188E−01(=)	1.7836E−04(−)	4.2259E−03(−)	5.2014E−01(=)
F19	Contest	3.0199E−11(+)	1.8368E−02(+)	8.1014E−10(+)	8.1527E−11(+)	3.6897E−11(+)	3.0199E−11(+)	3.0199E−11(+)
F20	Contest	1.7245E−06(+)	2.7065E−01(=)	4.9784E−09(−)	9.2930E−01(=)	1.5160E−03(−)	6.3503E−02(=)	1.1195E−01(=)
F21	Contest	3.0199E−11(+)	6.0459E−07(+)	5.0723E−10(+)	1.9527E−03(−)	4.4592E−04(+)	3.0199E−11(+)	3.0199E−11(+)
F22	Contest	3.0199E−11(+)	3.0199E−11(+)	3.0199E−11(+)	3.0199E−11(+)	3.8249E−09(+)	3.0199E−11(+)	3.0199E−11(+)
F23	Contest	3.0199E−11(+)	5.7460E−02(=)	3.0317E−02(−)	1.2362E−03(+)	1.5969E−03(+)	3.0199E−11(+)	3.0199E−11(+)
F24	Contest	1.2023E−08(+)	9.5207E−04(−)	1.0188E−05(−)	3.1830E−03(+)	2.6243E−03(+)	5.6073E−05(−)	1.8731E−07(+)
F25	Contest	1.3594E−07(+)	5.6073E−05(−)	8.1014E−10(+)	5.8945E−01(=)	3.2651E−02(+)	2.2273E−09(+)	6.6955E−11(+)
F26	Contest	1.0315E−02(+)	1.0666E−07(−)	1.7294E−07(−)	6.2027E−04(−)	7.7387E−06(−)	3.3242E−06(−)	3.9167E−02(−)
F27	Contest	1.2212E−02(+)	8.8411E−07(−)	3.5708E−06(−)	1.8608E−06(−)	6.7650E−05(−)	3.2555E−07(−)	8.1875E−01(=)
F28	Contest	1.4298E−05(+)	8.3520E−08(−)	3.0199E−11(−)	1.9963E−05(−)	7.1186E−09(−)	4.5043E−11(−)	4.5043E−11(−)
+ / = /-		28/0/0	19/3/6	16/2/10	19/4/5	19/1/8	19/2/7	20/5/3

**Figure 4:**
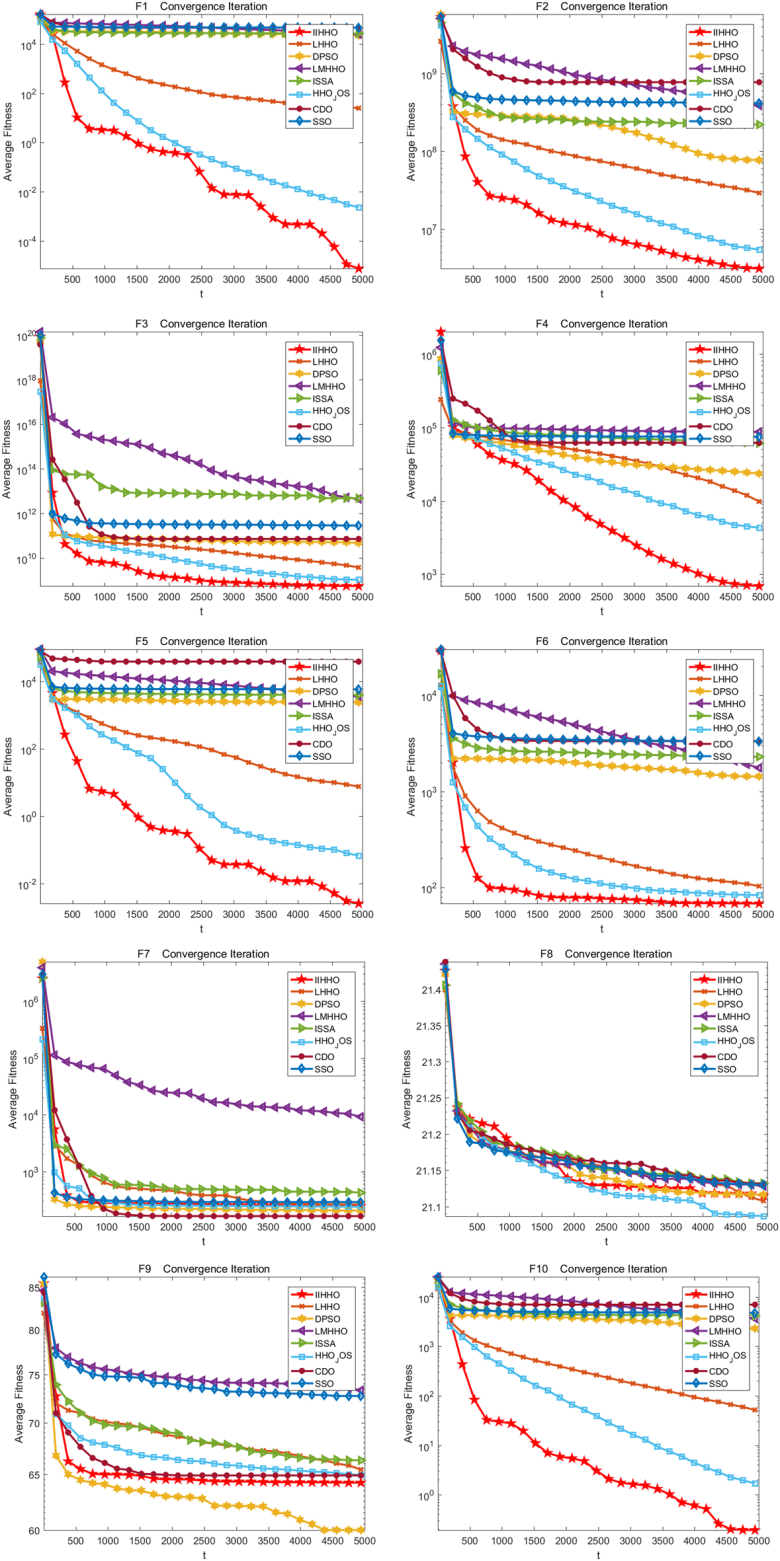

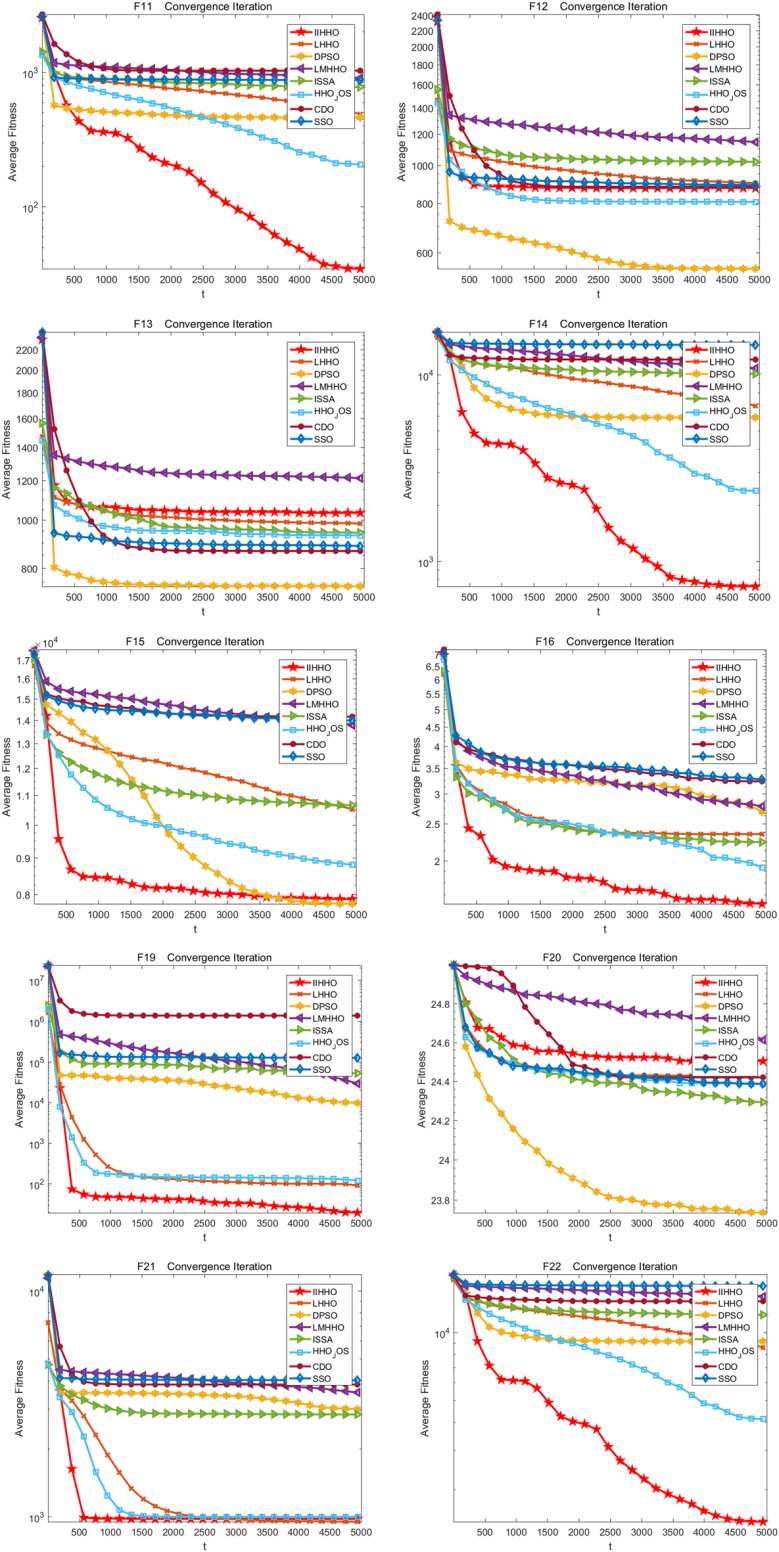

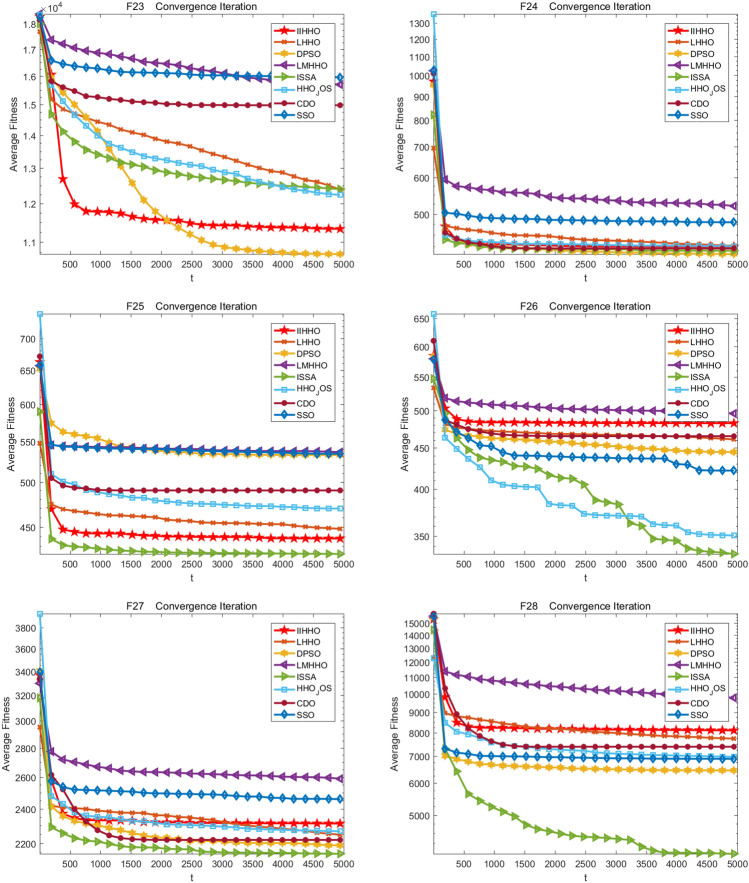
Iterative convergence graph of each improved algorithm.

The experimental effects of IIHHO are shown in Fig. 4. From the test results of unimodal functions F1 to F5 in Table 5, the optimal value, average value, worst value and standard deviation of IIHHO in functions F1, F3, F4 and F5 are the best, although the optimal value obtained by the algorithm in function F2 is slightly inferior to that of HHO_ JOS algorithm is still better than other algorithms. As shown in Fig. 4, the algorithm has the best iterative effect on all unimodal functions and can jump out of the local optimum to find a more accurate optimal solution. In general, IIHHO algorithm is conducive to solving unimodal functions. Then, the improvement of the algorithm on the basic multi-mode function is analyzed from Table 5. From F6 to F20, the results of IIHHO on the relevant parameters of functions F6, F10, F11, F14 and F19 are the best. Although the values of functions F8, F9, F12, F13 and F15 are not optimal, combined with Wilcoxon test, the difference of the optimal solution obtained by the algorithm is greater than most algorithms and the average value is better than most algorithms, proving that the results obtained by the algorithm are still better than most algorithms, which further proves that the algorithm can show superior performance in solving the basic multi-mode function. Finally, the improvement effect of the algorithm between F21 and F28 is analyzed. On the whole, the processing advantage of the algorithm is not big, but the proposed algorithm can still get more accurate optimal value, average value, maximum difference and standard deviation in function F22, and the average value in functions F21, F23 and F25 is still better than most algorithms. Combined with the Context value, the average ranking of the algorithm in 50 dimensions is 2.6429. In general, IIHHO algorithm has certain competitiveness in the comparison of seven variant algorithms, which also verifies that IIHHO algorithm has certain research value.

### Performance comparison between IIHHO and SOTA algorithms

The experimental results compared with the improved algorithm verify the performance of the algorithm. In this chapter, IIHHO is used to compared the data with the currently selected LSHADE_ SPACMA^41^, LSHADE_cnEpSin^42^ and EA4eig^43^ totally three state of the art (SOTA) algorithms, which means top ranking algorithms. The corresponding parameter settings are shown in Table 7. All algorithms are applied to CEC 2022^44^ for testing. Unlike CEC 2013, CEC 2022 has only 12 single objective test functions with boundary constraints, which are unimodal function (F1), multimodal function (F2–F5), mixed function (F6–F8) and combined function (F9-F12), and the test dimension selection is only 2 dimensions, 10 and 20 dimensions. This paper selects 20 dimensions as the problem scale, and Wilcoxon test is also performed, which is represented by the value of Context parameter. The experimental results are shown in Tables 8 and 9.

**Table 7:** Parameter setting for each SOTA algorithm.

Algorithms	Parameters
LSHADE_SPACMA	$p\_best\_rate=0.11$*;*$arc\_rate=1.4$*;*$memory\_size=5$*;*$min\_pop\_size=4.0$*;*$p\_First\_class\_percentage=0.5$
LSHADE_cnEpSin	$p\_best\_rate=0.11$*;*$arc\_rate=1.4$*;*$memory\_size=5$*;*$min\_pop\_size=4.0$
EA4eig	$myeps={e}^{-6}$*;*$pmax=0.25$

**Table 8:** Optimization results of SOTA algorithm in 50 dimensions.

F	Index	LSHADE_SPACMA	LSHADE_cnEpSin	EA4eig	IIHHO
F1	Best	**0.0000E+00**	**0.0000E+00**	**0.0000E+00**	1.5758E−05
	Aver	**0.0000E+00**	**0.0000E+00**	**0.0000E+00**	1.7069E+01
	Worst	**0.0000E+00**	**0.0000E+00**	**0.0000E+00**	5.0106E+02
	Std	**0.0000E+00**	**0.0000E+00**	**0.0000E+00**	9.1425E+01
	Rank	1	1	1	2
F2	Best	4.4896E+01	4.1091E+00	**0.0000E+00**	7.7936E−02
	Aver	4.8945E+01	2.1359E+01	**5.3155E−01**	4.8910E+01
	Worst	4.9085E+01	3.7655E+01	**3.9866E+00**	9.4927E+01
	Std	7.6481E+00	7.5746E+00	**1.3784E+00**	1.7425E+01
	Rank	4	2	1	3
F3	Best	**0.0000E+00**	0.0000E+00	0.0000E+00	2.4162E+01
	Aver	**0.0000E+00**	6.8212E−14	7.9581E−14	3.9186E+01
	Worst	**0.0000E+00**	1.1369E−13	1.1369E−13	6.8384E+01
	Std	**0.0000E+00**	5.6647E−14	5.2988E−14	1.0003E+01
	Rank	1	2	3	4
F4	Best	**0.0000E+00**	3.9799E+00	1.9899E+00	4.3778E+01
	Aver	**2.8522E+00**	5.7709E+00	9.4521E+00	8.3643E+01
	Worst	**5.9698E+00**	7.9598E+00	1.6914E+01	1.1740E+02
	Std	1.4719E+00	**1.2639E+00**	3.3942E+00	1.5155E+01
	Rank	1	2	3	4
F5	Best	**0.0000E+00**	**0.0000E+00**	**0.0000E+00**	9.2252E+02
	Aver	**0.0000E+00**	**0.0000E+00**	**0.0000E+00**	1.4045E+03
	Worst	**0.0000E+00**	**0.0000E+00**	**0.0000E+00**	1.8203E+03
	Std	**0.0000E+00**	**0.0000E+00**	**0.0000E+00**	2.3449E+02
	Rank	1	1	1	2
F6	Best	1.4809E+00	2.2571E+00	**2.0834E−02**	1.4672E+02
	Aver	9.6543E+00	7.2287E+00	**3.1220E−01**	4.0607E+03
	Worst	2.1172E+01	1.9096E+01	**1.2416E+00**	1.8591E+04
	Std	5.7276E+00	4.2923E+00	**3.6364E−01**	5.9499E+03
	Rank	3	2	1	4
F7	Best	3.1217E−01	0.0000E+00	**0.0000E+00**	6.2398E+01
	Aver	1.3863E+01	6.3778E+00	**4.2917E+00**	1.3880E+02
	Worst	2.2599E+01	2.0995E+01	**2.1307E+01**	2.0559E+02
	Std	9.6595E+00	9.2715E+00	**5.7563E+00**	4.2123E+01
	Rank	3	2	1	4
F8	Best	4.1329E−01	1.6448E−01	**7.6628E−02**	2.5559E+01
	Aver	1.9353E+01	**1.3196E+01**	1.7522E+01	4.1353E+01
	Worst	2.0829E+01	2.0607E+01	**2.0564E+01**	2.4771E+02
	Std	**3.7304E+00**	9.1373E+00	6.0456E+00	3.9811E+01
	Rank	3	1	2	4
F9	Best	1.8078E+02	1.7764E+02	**1.6534E+02**	1.8083E+02
	Aver	1.8078E+02	1.7925E+02	**1.6534E+02**	1.8221E+02
	Worst	1.8078E+02	1.8032E+02	**1.6534E+02**	1.8946E+02
	Std	8.6723E−14	7.0824E−01	**0.0000E+00**	2.1694E+00
	Rank	3	2	1	4
F10	Best	1.0021E+02	1.0022E+02	1.0020E+02	**4.9318E+01**
	Aver	**1.0025E+02**	1.0026E+02	1.0027E+02	7.3804E+02
	Worst	**1.0032E+02**	1.0034E+02	1.0034E+02	2.5579E+03
	Std	**2.4261E−02**	2.9566E−02	3.3874E−02	6.0845E+02
	Rank	1	2	3	4
F11	Best	3.0000E+02	3.0000E+02	3.0000E+02	**2.1606E−02**
	Aver	3.1333E+02	**3.0667E+02**	3.1667E+02	3.3656E+02
	Worst	4.0000E+02	**4.0000E+02**	4.0000E+02	5.4744E+02
	Std	3.4575E+01	**2.5371E+01**	3.7905E+01	9.8759E+01
	Rank	2	1	3	4
F12	Best	2.3069E+02	2.3068E+02	**1.8946E+02**	2.7011E+02
	Aver	2.3516E+02	2.3614E+02	**1.9956E+02**	4.0097E+02
	Worst	2.4302E+02	2.4565E+02	**2.0000E+02**	6.4740E+02
	Std	3.2509E+00	4.5366E+00	**1.9256E+00**	8.2494E+01
	Rank	2	3	1	4
Total rank		2.0833	1.7500	1.7500	3.5833

The minimum values under the same parameter comparison are in bold.

**Table 9:** Wlicoxon test results of each improved algorithm.

F	Index	LMHHO	ISSA	DPSO
F1	Contest	1.2118E−12(+)	1.2118E−12(+)	1.2118E−12(+)
F2	Contest	2.1778E−05(+)	1.5581E−08(+)	6.3018E−12(+)
F3	Contest	1.2118E−12(+)	1.3369E−11(+)	8.8675E−12(+)
F4	Contest	2.8773E−11(+)	3.0199E−11(+)	2.9468E−11(+)
F5	Contest	1.2118E−12(+)	1.2118E−12(+)	1.2118E−12(+)
F6	Contest	3.0199E−11(+)	3.0199E−11(+)	3.0199E−11(+)
F7	Contest	3.0199E−11(+)	2.9155E−11(+)	2.9766E−11(+)
F8	Contest	3.0199E−11(+)	3.0199E−11(+)	3.0199E−11(+)
F9	Contest	1.2118E−08(+)	3.0199E−11(+)	1.2118E−12(+)
F10	Contest	5.5727E−10(+)	5.5727E−10(+)	5.5727E−10(+)
F11	Contest	1.1636E−07(+)	2.673E−09(+)	6.2825E−07(+)
F12	Contest	3.0199E−11(+)	3.0199E−11(+)	3.0199E−11(+)
+/=/−		12/0/0	12/0/0	12/0/0

Comparing with the SOTA algorithm in Table 8, it is found that although the values obtained by the proposed IIHHO algorithm do not have obvious advantages, and the average ranking obtained is 3.5833, it has no obvious competitiveness compared with the other three SOTA algorithms, and the optimal value obtained by the proposed algorithm in functions F10 and F11 is better than the three SOTA algorithms, and the optimal value in function F2 is better than LSHADE_SPACMA and LSHADE_cnEpSin, which proves that the algorithm still has certain advantages in the performance of jumping out of the local optimum to get a more accurate optimal value.

## Performance of IIHHO on engineering applications

In this section, two different engineering benchmark problems—welding beam design problem, pressure vessel design problem, and threE−bar truss design problem are used to evaluate the performance of IIHHO in practical problems, and the IIHHO algorithm is compared with LMHHO, ISSA, DPSO, LHHO, HHO_JOS, CDO, SSO totally 7 algorithms are applied together in the problem for comparison, and the comparison results are obtained.

### Welded beam engineering design problem

**Figure 5:**
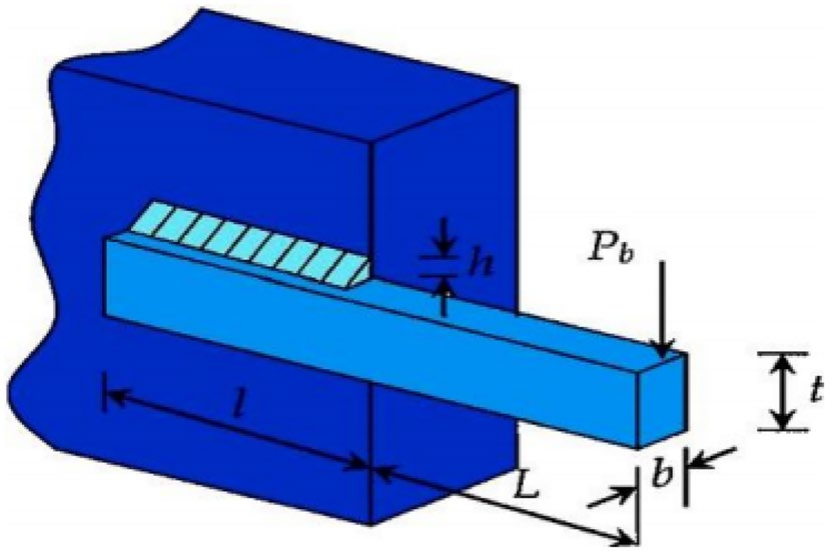
Example of welding beam design^47^.

The well-known welded beam problem is a typical engineering design problem proposed by Ashutosh and Vikram^45^ . The corresponding example is shown in Fig. 5. The purpose is to find the best design to minimize the manufacturing price of welded beams under multiple constraints. The parameters needed in the design of welded beam include the thickness (*b*), length (*L*), weld thickness (*h*) and reinforcement height (*t*). The eight algorithms are applied to the welded beam problem for comparison. According to the results in Table 10, it is found that using IIHHO algorithm can obtain the minimum production cost of 1.72493, which is second only to DPSO.

**Table 10:** Comparison results of welding beam design problems.

Algorithms	Optimal values of parameters				Optimal cost
	*h*	*L*	*t*	*b*	
IIHHO	0.20567	**3.47168**	9.03661	0.20573	1.72493
LMHHO	0.18462	3.87114	9.30761	0.20468	1.75621
ISSA	**0.10000**	3.72361	9.75684	0.10000	1.72801
DPSO	0.24530	3.66759	9.40436	0.21435	**1.72486**
LHHO	0.20374	3.51394	9.03662	0.20573	1.72760
HHO_JOS	0.20087	3.64613	**9.02094**	0.20645	1.74357
CDO	0.19416	3.76059	9.05141	0.20790	1.75607
SSO	0.18846	3.63018	10.00000	**0.20185**	1.77958

The minimum values under the same parameter comparison are in bold.

### Pressure vessel engineering design problem

**Figure 6:**
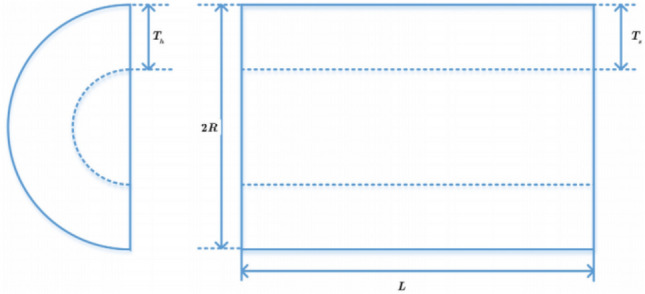
Example of pressure vessel design^46^.

The pressure vessel design problem is an engineering design problem to minimize the production cost of pressure vessels. The corresponding example is shown in Fig. 6. *L* is the section length of the cylinder part, *2R* is the inner wall diameter of the cylinder, *Th* and *Ts* represent the wall thickness of the head and the wall thickness of the circular cylinder respectively. These four indicators are the four optimization variables of the pressure vessel problem. The IIHHO algorithm is also applied to the welded beam problem. The experimental results are shown in Table 11. It is found that IIHHO algorithm has obvious optimization effect on variables, and can achieve the optimal cost of 5887.74947.

**Table 11:** Comparison results of pressure vessel design problems.

Algorithms	Optimal values of parameters				Optimal cost
	*Ts*	*Th*	*R*	*L*	
IIHHO	0.77958	**0.38535**	**40.39271**	198.98501	**5887.74947**
LMHHO	1.14042	0.56874	56.21543	55.58639	7015.40377
ISSA	25.31287	24.76300	81.57112	200.00000	5929.09177
DPSO	1.68907	0.77316	42.35464	182.65238	5987.27063
LHHO	**0.64285**	0.41794	43.67079	158.08052	6009.81495
HHO_JOS	1.15646	0.56801	59.53565	**37.005165**	5958.37415
CDO	0.80096	0.40804	41.08562	189.66377	5979.73895
SSO	0.87116	0.47080	44.05068	158.85328	6159.11384

The minimum values under the same parameter comparison are in bold.

## Conclusion

The original HHO algorithm is a kind of algorithm with certain research value. Because of its strong global search ability and less conditional parameters, it has been used to solve practical problems in recent years. The algorithm also has the disadvantages of poor search performance when solving low dimensional problems and easy to fall into local optimum after searching. Therefore, this paper improves the HHO algorithm by adjusting the intermittent energy factor, the attenuation vector and the flight step adjusted by Cardano function to improve the local search ability and calculation accuracy of the algorithm. On the CEC 2013 function test set, the improved IIHHO has obvious optimization effect on 50 dimensional unimodal function and basic multimode function, and the convergence speed is faster, and the results are better. Compared with three SOTA algorithms in CEC 2022, IIHHO also shows certain optimization effect, which proves that the improved IIHHO algorithm has strong stability and robustness. Finally, the algorithm is applied to two classical engineering problems to test its performance; Although the improvement effect of IIHHO algorithm is stable, it still has some problems. For example, compared with SOTA algorithm, there are no advantages in the average and other related parameters; The results of solving composite function in CEC 2013 are poor; The intermittent time of energy recovery is too long. Therefore, some adjustments can be made in the future research:

1. The cycle mechanism of energy can be modified. First, it is considered to appropriately shorten the rest time of prey so that the exploration activities can still improve the global search ability in the early cycle. Second, it is further considered to carefully consider the recovery level of energy in combination with the actual situation.
2. The variance update formula of Levy flight function can be further improved. Firstly, the influence of random value should be appropriately reduced. Secondly, the classical variance structure should be decomposed. There is also a certain research value for the design of parameters *u* and *v*.
3. The application field of the improved algorithm is still limited to classical engineering problems, so it is necessary to constantly explore new fields to meet the needs of real life and production.

## Data Availability

All data for this study are available from the corresponding author.
