# Author Correction: Integrated improved Harris hawks optimization for global and engineering optimization

**DOI:** 10.1038/s41598-024-60232-1

**Published:** 2024-05-02

**Authors:** Chengtian Ouyang, Chang Liao, Donglin Zhu, Yangyang Zheng, Changjun Zhou, Taiyong Li

**Affiliations:** 1 School of Information Engineering, Jiangxi University of Science and Technology, Ganzhou, 341000 China; 2 School of Computer Science and Technology, Zhejiang Normal University, Jinhua, 321004 China; 3 School of Computing and Artificial Intelligence, Southwestern University of Finance and Economics, Chengdu, 611130 China

Correction to: *Scientific Reports* 10.1038/s41598-024-58029-3, published online 28 March 2024

The original version of this Article contained an error in the spelling of the author Chengtian Ouyang which was incorrectly given as Chentian Ouyang.

The original Article has been corrected.

